# The Mechanism of Ultrasonic Lysis of *Enterococcus faecium* F11.1G in Repairing LPS-Induced Inflammatory Damage in IECs via RNA-seq and LC-MS

**DOI:** 10.3390/cells15020103

**Published:** 2026-01-06

**Authors:** Tiantian Bai, Yanlong Zhang, Guangxu E, Meng Zhang, Xuefeng Guo, Junfeng Liu

**Affiliations:** 1 College of Life Science and Technology, Tarim University, Alar 843300, China; 2 Xinjiang Production & Construction Corps Key Laboratory of Protection and Utilization of Biological Resources in Tarim Basin, Alar 843300, China; 3 College of Animal Science and Technology, Tarim University, Alar 843300, China; 4 Key Laboratory of Livestock and Grass Resources Utilization Around Tarim, Ministry of Agriculture and Rural Areas (Co-Construction by Ministries and Provinces), Alar 843300, China

**Keywords:** *Enterococcus faecium*, LPS, metabolomics, transcriptomics, inflammation, NF-κB, MAPK

## Abstract

**Highlights:**

**What are the main findings?**

- Ultrasonicated *Enterococcus faecium* F11.1G effectively alleviates the LPS-induced inflammatory damage in intestinal epithelial cells.
- 10^8^ CFU/mL F11.1G significantly suppresses the excessive secretion of pro-inflammatory factors (*IL-6*, *IL-8*, *IL-1β*, and *TNF-α*).
- The anti-inflammatory mechanism of F11.1G primarily involves inhibiting the activation of the NF-κB/MAPK signaling pathways and may synergistically regulate the purine/endocannabinoid metabolic networks.

**What are the implications of the main findings?**

- These findings provide experimental evidence supporting the potential of ultrasonicated *Enterococcus faecium* F11.1G as a postbiotic agent for alleviating intestinal inflammation.
- The study suggests that F11.1G exerts its anti-inflammatory effects through multi-target modulation of inflammatory signaling pathways and metabolic networks.

**Abstract:**

Lipopolysaccharide (LPS)-induced damage to the intestinal epithelial barrier leads to gut inflammation, and intracellular metabolites of lactic acid bacteria may participate in regulating this process to exert probiotic effects. This study aimed to investigate the repair effects and molecular mechanisms of ultrasonic disruption-treated *Enterococcus faecium* F11.1G (F11.1G) on the model (primary lamb IECs + 5 μg/mL LPS for 6 h). Then, results demonstrated that 10^8^ CFU/mL F11.1G significantly suppressed the excessive secretion of pro-inflammatory factors (*IL-6*, *IL-8*, *IL-1β*, *TNF-α*) induced by LPS. Gene Ontology (GO) analysis revealed that differentially expressed genes (DEGs) were primarily enriched in cellular response to lipopolysaccharide, inflammatory response, and canonical NF-κB signaling pathways. Kyoto Encyclopedia of Genes and Genomes (KEGG) analysis showed enrichment in NF-κB signaling pathway and MAPK signaling pathway. PPI network identified key genes including *IL-1β*, *TNF*, *IL-8*, *RELB*, *FOS*, *TNFAIP3*, *NFKBIA*, and *MMP9*. KEGG analysis indicated differentially abundant metabolites (DAMs) enrichment in purine metabolism and the endocannabinoid system. Spearman correlation analysis revealed positive correlations between pro-inflammatory genes and endogenous protective metabolites, such as adenosine and PEA, while showing negative correlations with multiple purine metabolites. Correlational analysis indicates that F11.1G alleviates intestinal inflammatory damage primarily by suppressing NF-κB/MAPK pathway activation and may synergistically regulate purine and endocannabinoid metabolism.

## 1. Introduction

Diarrhea is one of the most common diseases in young livestock, with enterotoxigenic *Escherichia coli* (ETEC) identified as the primary pathogen responsible for lamb diarrhea [1]. ETEC adheres to intestinal epithelial cells (IECs), leading to the production and replication of enterotoxin [2]. Clinical reports indicate that ETEC infection exhibits intestinal pathogenicity, causing severe clinical symptoms such as vomiting and diarrhea, along with increased morbidity and mortality in young animals [3]. As a key organ for nutrient absorption and immune defense, the intestinal barrier structure comprises a monolayer of IECs. This structure not only functions as a physical barrier but also actively participates in immune surveillance and regulation [4]. Tight junctions (TJs) between adjacent IECs form a selectively permeable barrier that facilitates nutrient absorption while effectively preventing the invasion of harmful microorganisms and toxins [5]. Lipopolysaccharide (LPS) originating from *Escherichia coli* (*E. coli*) constitutes a primary component of Gram-negative bacterial cell walls and is released through sloughing or bacterial lysis; it has been demonstrated to be a key factor influencing intestinal barrier function [6]. Research indicates that LPS can activate pro-inflammatory cytokines such as *interleukin-6* (*IL-6*) and *tumor necrosis factor-alpha (TNF-α*), while damaging the intercellular TJs between IECs [7], thereby inducing intestinal inflammation. Notably, chronic inflammation can precipitate a spectrum of clinical manifestations, ranging from mild gastrointestinal dysfunction to severe malignant lesions [8]. Currently, widely used anti-inflammatory drugs have drawn attention due to their potential to cause significant adverse reactions, including gastrointestinal risks [9]. Against this backdrop, probiotics are a potential alternative biological agent in the prevention and alleviation of inflammation [10]. Their advantage lies in reducing adverse reactions, such as gut dysbiosis, that may be induced by other anti-inflammatory drugs [11]. This also influences mucosal immune responses and inflammatory processes by regulating cell proliferation and immune responses [12]. Among these, *Enterococcus faecium* CFR 3003 has been demonstrated to exhibit anti-inflammatory effects [13]. Moreover, *Enterococcus faecalis* KUMS-T48 downregulates *Interleukin-1 beta (IL-1β)* and upregulates *Interleukin-10 (IL-10)* in human colon adenocarcinoma cells (HT-29), confirming its significant anti-inflammatory effects [14]. However, the efficacy of novel or specific Enterococcus strains requires empirical validation.

The *Enterococcus faecium* F11.1G, isolated by our research team from Xinjiang yeast starter, inhibits the growth of multiple pathogenic bacteria [15] and regulates the rumen microbiota [16]. Its genome contains diverse secondary metabolite synthesis gene clusters with a well-defined intracellular metabolite profile. It exhibits close phylogenetic affinity to the genome of the probiotic strain T110 (GenBank no. CP006030), displaying high similarity yet structural variations [17]. Unlike conventional studies using live or heat-inactivated bacteria, this research employs ultrasonic disruption to prepare intact intracellular metabolites as a postbiotic formulation, aiming to investigate the synergistic mechanisms of multiple natural metabolites. Based on the aforementioned studies, we propose the following hypothesis: Intracellular metabolites (e.g., polysaccharides, lipids, hormones) obtained from *Enterococcus faecium* F11.1G via ultrasonic disruption may exert a restorative effect in LPS-induced intestinal inflammation by modulating the NF-κB/MAPK signaling pathway. However, whether *Enterococcus faecium* F11.1G metabolites can repair LPS-induced inflammation in IECs remains unknown. This study aims to elucidate the gene expression, metabolite alterations, and their synergistic regulatory networks involving F11.1G in the repair of inflammatory damage by integrating cellular experiments with transcriptomic and metabolomic analyses.

## 2. Materials and Methods

### 2.1. Cell Isolation, Identification and Growth Curve Determination

IECs were isolated from the lamb duodenum following the method of Li et al. (2017) [18]. The cells were cultured in DMEM/F12 medium (Cellverse Co., Ltd., PriMed-iCell-001, Shanghai, China) containing 10% fetal bovine serum (Cellverse Co., Ltd., iCell-0500-1, China) and 1% penicillin–streptomycin (Cellverse Co., Ltd., iCell-15140-122, China) at 37 °C in a 5% CO_2_ incubator (Eppendorf, C170, Hamburg, Germany).

For immunofluorescence (IF), cells were seeded onto coverslips in 6-well plates at a density of 2 × 10^4^ cells per well and cultured until confluence. Subsequent experiments were conducted in accordance with Wang et al. (2019) [19]. Briefly, the cells were washed three times with ice-cold phosphate-buffered saline (PBS) and fixed with precooled 4% paraformaldehyde at 4 °C for 15 min. Following fixation, the cells were rewashed with PBS. The coverslips were then incubated with 50–100 μL of permeabilization working solution at room temperature for 20 min, and subsequently washed three times for 5 min each with PBS on a decolorizing shaker. Non-specific binding sites were blocked by covering the cells with 3% bovine serum albumin (BSA) (Solarbio, S9070, Beijing, China) for 30 min at room temperature. The cells were subsequently incubated overnight at 4 °C in a humidified chamber with primary antibodies; the primary antibodies were against Cytokeratin 19 (CK-19) (rabbit, 1:200 dilution, Proteintech, 10712-1-AP, Rosemont, IL, USA). The following day, the coverslips were washed three times with PBS (5 min per wash) on a shaker and incubated with the secondary antibody against Alexa Fluor 488-conjugated Goat anti-Rabbit IgG (H + L) (1:500 dilution, Proteintech, SA00006-2, USA), for 50 min at room temperature in the dark. After another three 5 min washes with PBS, the coverslips were slightly dried, and the cell nuclei were stained with DAPI (1:1000 dilution, Solarbio, C0060, China) for 10 min at room temperature in the dark. A final series of three 5 min PBS washes was performed. The coverslips were then mounted onto glass slides using an anti-fade mounting medium. Fluorescence images were captured immediately using an inverted fluorescence microscope (Nikon DS-Ri2, Tokyo, Japan) with a 200× objective lens. The following filter sets were used for detection: DAPI (excitation 330–380 nm, emission 420 nm, blue for nuclei), FITC/Alexa Fluor 488 (excitation 465–495 nm, emission 515–555 nm, green for positive signal), and Cy3 (excitation 510–560 nm, emission 590 nm, red for positive signal).

Cells were seeded at 2 × 10^4^ cells per well in a 96-well cell culture plate and cultured in a 37 °C, 5% CO_2_ incubator. Cell viability was assessed on days 1, 2, 3, 4, 5, 6, and 7 using a 10% (*v*/*v*) CCK-8 (Sigma-Aldrich Co., Taufkirchen, Germany) solution. Cell-free medium was used as the blank control. Wells were gently mixed and incubated in the incubator, protected from light, for 2 h. The optical density (OD value) at 450 nm was measured for each group using a microplate reader (BioTek Instruments, H1, Winooski, VT, USA).

Cell viability was calculated using the following formula:

Cell Viability (%) = [(OD_Experimental − OD_Blank)/(OD_NC − OD_Blank)] × 100%

The viability of the NC group was defined as 100%.

### 2.2. Establishment of a Cellular Inflammatory Injury Model

To assess the production of inflammatory cytokines, IECs were seeded in 96-well and 6-well plates at a density of 2 × 10^4^ cells/mL and cultured at 37 °C until reaching confluence. To determine the optimal conditions for inducing inflammatory injury, IECs were treated with LPS (Sigma-Aldrich Co., L2880, Germany) at concentrations of 0, 1, 5, 10, and 20 μg/mL for durations of 3, 6, 12, and 24 h. Subsequently, cell viability was determined by the CCK-8 assay, following the identical procedure detailed in Section 2.1. Simultaneously, the cell culture supernatant and total RNA were collected from the 6-well plates. The levels of pro-inflammatory cytokines/chemokines IL-6, Interleukin-8 (IL-8), IL-1β, and TNF-α and the anti-inflammatory cytokine IL-10 were analyzed using commercial enzyme-linked immunosorbent assay (ELISA) kits (Sheep, YL32272, YJ022106, YJ966713, YJ958471, YJ477826, Shanghai Enzyme-linked Biotechnology Co., Ltd., Shanghai, China) and RT-qPCR, respectively. RT-qPCR experiments were conducted in accordance with Sun et al. (2025) [20]. Total RNA was isolated from IECs using the TransZol Up Plus RNA kit (TransGen Biotech, ER501, Beijing, China). The concentration and purity of total RNA were assessed using a NanoDrop 8000 spectrophotometer (NanoDrop Technologies, Wilmington, DE, USA). The cDNA was synthesized using the Takara Reverse Transcription Kit (TaKaRa Bio, RR097A, Beijing, China) for qPCR assays. Each group of IECs was analyzed on a CFX96 real-time PCR Detection System (Bio-Rad Laboratories, Hercules, CA, USA). Each 15 µL of RT-qPCR reaction contained 7.5 µL of SYBR Green real-time PCR Mix, 5.5 µL of nuclease-free water, 0.5 µL of each primer (10 µM), and 1.0 µL of cDNA (500 ng/µL) (TransGen Biotech, S31025, China). The cycling conditions were as follows: 94 °C for 30 s; 40 cycles of 94 °C for 20 s, 58 °C for 15 s, and 72 °C for 15 s; followed by melt-curve analysis (95 °C for 15 s, 60 °C for 30 s, 95 °C for 15 s). Melt-curve parameters used the instrument’s default settings. Each sample was run in triplicate. The housekeeping gene encoding glyceraldehyde-3-phosphate dehydrogenase (GAPDH) was used to normalize the expression levels of target genes using the 2^−ΔΔCt^ method. Primers were designed as listed in Table 1.

### 2.3. Enterococcus faecium F11.1G and Cultivation Conditions

The *Enterococcus faecium* F11.1G used in this experiment was an independently isolated strain by Guo Xuefeng’s team at Tarim University and is stored at the China Center for Type Culture Collection (Wuhan, China) (accession number: M2020793). The strain was cultivated and maintained in De Man, Rogosa, and Sharpe (MRS) broth at 37 °C for 24 h under anaerobic conditions. By measuring the bacterial optical density (OD), 1 × 10^9^ CFU/mL (the standard number of viable probiotic cells) was chosen as the ready-to-use cell culture. The obtained fresh cultures were centrifuged at 8000× *g* at 4 °C for 15 min in a high-speed refrigerated centrifuge (Thermo Scientific, Waltham, MA, USA). Pre-cooled PBS was used to wash the bacterial precipitate 3 times. Then, ultrasonically crushed bacterial extracts were obtained by ultrasonic crushing in an ice bath for 15 min. The supernatant was collected by centrifugation (8000× *g*, 15 min) at 4 °C, passed through a 0.45-micron filter membrane and finally stored at −20 °C for future use. Ultrasonically disrupted *Enterococcus faecium* F11.1G will hereafter be referred to as F11.1G.

### 2.4. Cytotoxicity Assay of Ultrasonicated F11.1G

IECs were seeded in 96-well tissue culture plates at a density of 2 × 10^4^ cells/mL and cultured at 37 °C until confluence. The original culture medium was aspirated, and the cells were then treated with F11.1G at various concentrations (10^6^, 10^7^, 10^8^, and 10^9^ CFU/mL F11.1G in DMEM/F12 medium) for 3 or 6 h. NC group, which received no treatment, was included. Subsequently, cell viability was determined by the CCK-8 assay, following the identical procedure detailed in Section 2.1.

### 2.5. Repair of LPS-Induced Inflammatory Damage in IECs Following F11.1G Intervention

IECs were seeded in 6-well plates at a density of 2 × 10^4^ cells/mL and cultured at 37 °C until confluence. Firstly, the reparative effect of 10^6^, 10^7^, and 10^8^ CFU/mL F11.1G on LPS-induced IECs inflammatory damage was assessed via CCK-8 assay, employing the methodology detailed in Section 2.1. Based on the aforementioned results, the viability levels of bacterial strains suitable for subsequent experiments were screened. Thus, the optimal inflammatory concentration and exposure duration for LPS-induced inflammatory injury were determined to be 5 μg/mL LPS for 6 h (Section 2.2). Additionally, the repair concentration for F11.1G was established at 10^8^ CFU/mL for a treatment duration of 3 h. Then, the experiment was divided into three groups: control group (NC), treated with 5 μg/mL LPS for 6 h (LPS), and treated with 5 μg/mL LPS for 6 h, followed by 10^8^ CFU/mL F11.1G for 3 h (LPSF). After the treatments, the cell culture supernatant was collected and centrifuged to remove cellular debris for the analysis of secreted inflammatory cytokines. Concurrently, the cells were washed three times with cold PBS and then lysed to measure the intracellular cytokine levels.

### 2.6. Transcriptomic Analysis via RNA-seq

Cells from the NC, LPS, and LPSF groups were subjected to the treatments outlined in Section 2.5. Following the respective treatments, cells were washed three times with PBS and lysed in TRIzol reagent (Invitrogen Life Technologies, 15596018CN, Carlsbad, CA, USA), and the resulting lysates were transferred to cryovials for immediate snap-freezing in liquid nitrogen. They were then sent to Personalbio Technologies Co., Ltd. (Shanghai, China) for transcriptomic analysis. The sequencing method was identical to that reported in Cheng et al. (2025) [21]. The transcriptomic data reported in the current study have been deposited in the ScienceDB database (Date doi: 10.57760/sciencedb.32300).

Total RNA was isolated using the TRIzol Reagent (Invitrogen Life Technologies, 15596018CN, USA); after that, the concentration, quality, and integrity were determined using a NanoDrop 8000 spectrophotometer (NanoDrop Technologies, USA). Three micrograms of RNA were used as input material for the RNA sample preparation. Sequencing libraries were generated according to the following steps. Firstly, mRNA was purified from the total RNA using poly-T oligo-attached magnetic beads. Fragmentation was carried out using divalent cations under elevated temperature in the Illumina proprietary fragmentation buffer. First-strand cDNA was synthesized using random oligonucleotides and Super Script II. Second-strand cDNA synthesis was subsequently performed using DNA Polymerase I and RNase H. The remaining overhangs were converted into blunt ends via exonuclease/polymerase activities, and the enzymes were subsequently removed. After adenylation of the 3′ ends of the DNA fragments, Illumina PE adapter oligonucleotides were ligated to prepare for hybridization. To select cDNA fragments of the preferred length of 400–500 bp, the library fragments were purified using the AMPure XP system (Beckman Coulter, Beverly, CA, USA). DNA fragments with ligated adaptor molecules on both ends were selectively enriched using the Illumina PCR Primer Cocktail in a 15-cycle PCR reaction. The products were purified (AMPure XP system) and quantified with the Agilent high sensitivity DNA assay on a Bioanalyzer 2100 system (Agilent, Santa Clara, CA, USA). The sequencing libraries were then sequenced on NovaSeq 6000 platform (Illumina, San Diego, CA, USA) at Shanghai Personal Biotechnology Co., Ltd. (Shanghai, China).

### 2.7. Metabolomic Analysis by LC-MS

Cells from the NC, LPS, and LPSF groups were subjected to the treatments outlined in Section 2.5; the cell culture supernatants were collected and snap-frozen in liquid nitrogen, and then sent to Personalbio Technologies Co., Ltd. (China) for metabolomic analysis. Chromatographic conditions and mass spectrometric conditions were identical to those reported in Naveed et al. (2025) [22]. The metabolomic data reported in the current study have been deposited in the ScienceDB database (Date doi: 10.57760/sciencedb.32299).

Sample preparation procedure: take 1 mL of thawed and homogenized sample, rapidly freeze it in liquid nitrogen, then freeze-dry using a freeze-dryer. Add 1 mL of pre-chilled methanol/acetonitrile mixture (1:1, *v*/*v*) to the freeze-dried sample, vortex for 30 s, then place it in a −20 °C refrigerator for 30 min to obtain metabolites. Centrifuge at 4 °C and 12,000 rpm for 10 min. Remove 850 μL of supernatant for vacuum concentration and drying. Resuspend the dried material in 150 μL of 50% methanol solution containing 5 ppm 2-chlorophenylalanine, vortex for 30 s. Centrifuge at 12,000 rpm for 10 min, and transfer the supernatant, filter it through a 0.22 μm microporous membrane, and transfer the filtrate to a sample vial for analysis. Transfer 10–20 μL from each filtered sample, combine them to prepare quality control samples for subsequent monitoring of instrument stability and data reliability.

Chromatographic conditions: chromatographic separation was performed on an ACQUITY UPLC HSS T3 column (100 Å, 1.8 µm, 2.1 mm × 100 mm) maintained at 40 °C. The mobile phase flow rate was set at 0.4 mL/min, and the injection volume was 2 µL with the auto-sampler temperature held at 8 °C. For both positive and negative ionization modes, the mobile phase consisted of (A) 0.1% formic acid in water and (B) 0.1% formic acid in acetonitrile. The gradient elution program was optimized as follows: 0–1 min, 5% B; 1–4.7 min, 5–95% B; 4.7–6 min, 95% B; 6–6.1 min, 95–5% B; 6.1–8.5 min, 5% B for column re-equilibration.

Mass spectrometric conditions: mass spectrometry data were acquired in data-dependent acquisition (DDA) mode on a Thermo Orbitrap Exploris 120 mass spectrometer controlled by Xcalibur software (version 4.7, Thermo, Waltham, MA, USA). Analysis was carried out in both positive and negative ionization modes with a heated electrospray ionization (HESI) source. The key parameters were configured as follows: the spray voltage, 3.5 kV (positive) and −3.0 kV (negative); sheath gas flow rate, 40 arbitrary units (arb); auxiliary gas flow rate, 10 arb; capillary temperature, 320 °C; and auxiliary gas heater temperature, 300 °C. The full MS scans were acquired across a mass range of *m*/*z* 70–1000 at a resolution of 60,000. The automatic gain control (AGC) target was set to ‘Standard’, with a maximum injection time (Max IT) of 100 ms. The top four most intense ions from the full scan were selected for fragmentation, with a dynamic exclusion duration of 4 s. Tandem MS/MS scans were performed at a resolution of 15,000 using higher-energy collisional dissociation (HCD) with a normalized collision energy of 30%. The AGC target for MS/MS was set to ‘Standard, with the Max IT configured to ‘Auto’.

Metabolite identification and data processing: Raw data obtained post-instrumentation were imported into the metabolomics analysis software MS-DIAL (version 4.9.221218), where they underwent sequential processing comprising peak extraction, peak alignment, noise reduction, missing value imputation, and normalization. The specific steps were as follows: First, chromatographic peaks not detected in more than 50% of quality control samples were filtered out. Subsequently, the software’s built-in gap-filling algorithm was employed to fill the remaining missing values, followed by data normalization to eliminate systematic errors. Metabolite structural identification was performed using the Passenot PSNGM metabolomics database. This repository integrates multi-source MS data from multiple repositories, including an in-house standard library, mzCloud, LIPIDMAPS, HMDB, MoNA, NIST_2020_MSMS, and an AI-predicted MS/MS spectral library. Key search parameters were configured as follows: MS1 mass deviation of 0.01 Da, MS2 mass deviation of 0.05 Da, smoothing level of 3, minimum peak height of 10,000, minimum peak width of 5, mass slice width of 0.05, and identification score threshold of 70.

### 2.8. IF Staining

Cells from the NC, LPS, and LPSF groups were subjected to the treatments outlined in Section 2.5. Cells grown on 24 mm coverslips in 6-well plates were processed for IF (proceed as outlined in Section 2.1). Primary antibodies: anti-phospho-p65 (rabbit, 1:2000 dilution, BIOSS, BS-23303R, Beijing, China), and anti-phospho-p38 (rabbit, 1:5000 dilution, Service-bio, GB153880, Wuhan, China), secondary antibody was horseradish peroxidase (HRP)-conjugated goat anti-rabbit (1:5000 dilution, Service-bio, GB23303, China), cell nuclei were stained with DAPI (1:1000 dilution, Proteintech, USA). The following filter sets were used for detection: DAPI (excitation 330–380 nm, emission 420 nm, blue for nuclei), FITC/Alexa Fluor 488 (excitation 465–495 nm, emission 515–555 nm, green for positive signal), and Cy3 (excitation 510–560 nm, emission 590 nm, red for positive signal).

### 2.9. Western Blot (WB) Analysis

Cells from the NC, LPS, and LPSF groups were subjected to the treatments outlined in Section 2.5. Subsequent experiments were conducted in accordance with Kiatsoonthon et al. (2024) [23]. Primary antibodies: anti-TLR4 (rabbit, 1:1000 dilution, Service-bio, GB11519, China), anti-MyD88 (rabbit, 1:1000 dilution, Proteintech, 23230-1-AP, USA), anti-p65 (rabbit, 1:1000 dilution, Service-bio, GB11997, China), anti-phospho-p65 (rabbit, 1:1000 dilution, BIOSS, BS-23303R, China), anti-p38 (rabbit, 1:1000 dilution, BIOSS, BS-0637R, China), anti-phospho-p38 (rabbit, 1:1000 dilution, Service-bio, GB153880, China), and anti-β-actin (1:5000 dilution, Service-bio, GB15003, China), secondary antibody was HRP-conjugated goat anti-rabbit (sheep, 1:5000 dilution, Service-bio, GB23303, China). The grayscale value was quantified using ImageJ software (version 1.54q, National Institutes of Health, Bethesda, MD, USA). The relative densitometric expression level of each target protein was normalized to that of β-actin.

### 2.10. Statistical Analysis

Statistical analyses were performed using the SPSS software package (SPSS 12.0, SPSS Inc., Chicago, IL, USA). One-way analysis of variance was performed and the significance of each mean value was determined by Tukey’s multiple range test. Technical replicates were averaged per biological replicate for statistical analysis. *p*  <  0.05 was considered to indicate statistical significance. Transcriptomics data and metabolomics data were analyzed on the online platform of PAISEEK (https://www.genescloud.cn (accessed on 14 November 2025)). The adjusted *p*-value of RNA-seq data was calculated using DESeq2, followed by a false discovery rate (FDR) correction. Only those genes showing a corrected *p*-value < 0.05 were retained for downstream analysis. Gene Ontology (GO) and pathway enrichment analyses of differentially expressed genes (DEGs) were performed using the Database for Annotation, Visualization and Integrated Discovery (DAVID). Specifically, GO biological processes (GO-BP) and KEGG pathway analyses were carried out to elucidate the biological functions of the common targets. Next, a protein–protein interaction (PPI) network was generated using the STRING database (version 12.0, https://cn.string-db.org); the species selected is ‘Ovis aries’, with the medium confidence level set to 0.400. Cytoscape (version 3.10.0) and the CytoHubba plugin were used to identify hub genes. Hub genes were determined by the intersection of the top 22 highly connected genes identified through four algorithms: Nodes, Edges, Degree, *p*-value. Metabolomic data were analyzed using *p* < 0.05, fold change (FC) ≥ 1, and VIP ≥ 1 as screening criteria; then, differentially abundant metabolites (DAMs) were further filtered using criteria of FDR < 0.01, fold change (FC) > 1, and VIP > 1, followed by intra-group metabolite correlation analysis.

## 3. Results

### 3.1. Isolation and Identification of Lamb IECs and Determination of Growth Curves

**Figure 1:**
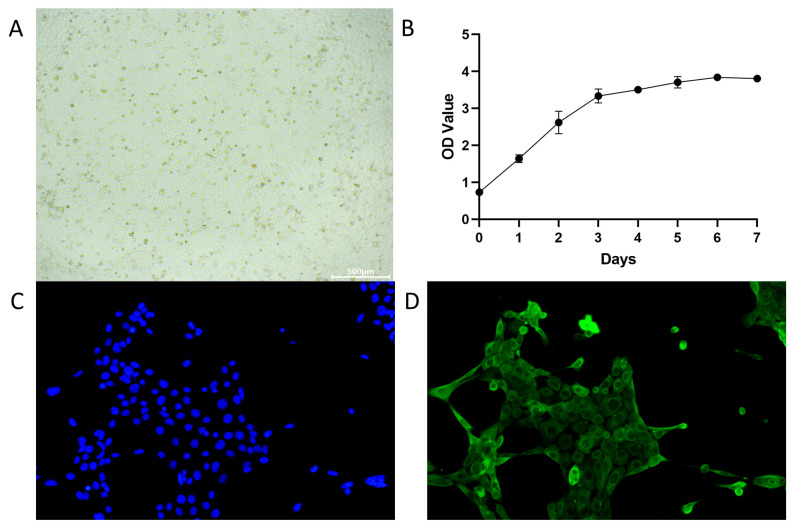
Morphology, isolation, identification, and growth curve of IECs. (**A**) Morphology of primary IECs (20× magnification). (**B**) Growth curve of IECs. Cell viability was assessed using the CCK-8 assay. (**C**) Nuclear staining with DAPI (200× magnification), with nuclear stained blue. (**D**) IF identification using the specific marker of CK-19 (200× magnification), with cells stained green. *n* = 3 independent IEC isolations.

The epithelial cells of the lamb small intestine exhibited a polygonal or oval morphology, arranged in a cobblestone-like pattern. As shown in Figure 1, the identified cells emitted green fluorescence under fluorescence microscopy. All cells were positive for the CK-19 antigen, confirming their identify as IECs. The growth curve indicated that IECs entered a logarithmic growth phase between days 0 and 3, during which they proliferated rapidly. From days 4 to 7, they entered a growth-inhibited phase characterized by slowed cell growth.

### 3.2. Establishment of Cellular Inflammatory Injury Models

**Figure 2:**
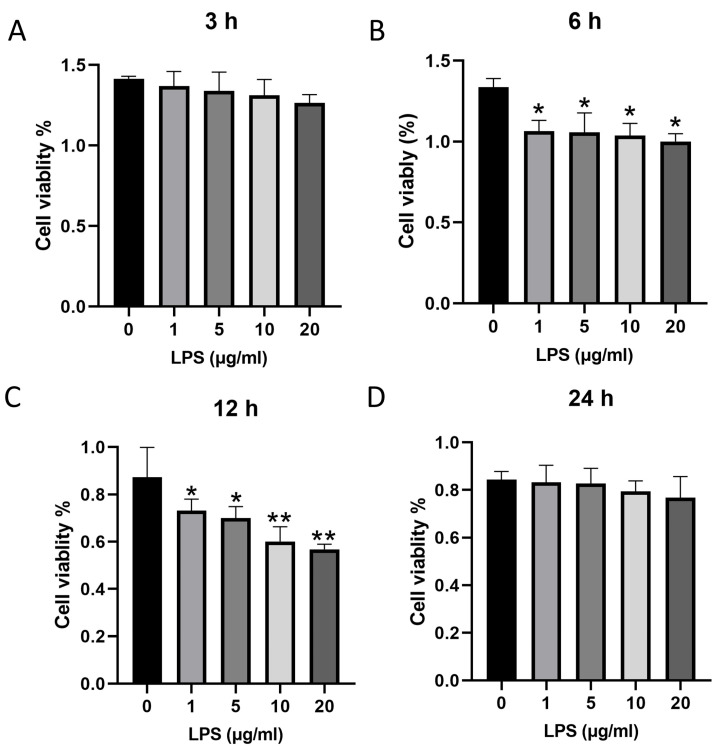
Effect of LPS on the viability of IECs. Panels showed cell viability following treatment with LPS for 3 h (**A**), 6 h (**B**), 12 h (**C**), and 24 h (**D**). * *p* < 0.05, ** *p* < 0.01 compared to the 0 μg/mL LPS. Data are presented as mean ± SD (*n* = 3 independent IEC isolations). Statistical significance was determined by one-way ANOVA with Tukey’s post hoc test for multiple comparisons.

As shown in Figure 2, LPS treatment significantly suppressed intestinal epithelial cell viability in a time-dependent manner. CCK-8 assays revealed that cell growth was markedly inhibited at 6 and 12 h post-treatment with LPS at concentrations of 1, 5, 10, and 20 μg/mL (*p* < 0.05), whereas no significant effects were detected at 3 and 24 h (*p* > 0.05).

### 3.3. Analysis of Inflammatory Factor Expression by RT-qPCR

**Figure 3:**
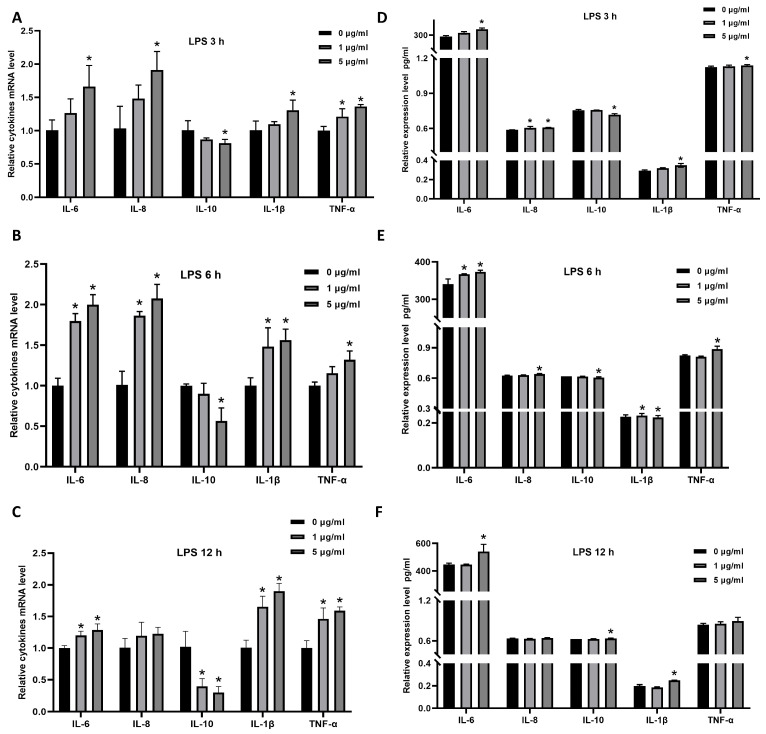
Effects of LPS on inflammatory cytokine production in IECs. mRNA levels of inflammatory cytokines in IECs treated with LPS for 3 h (**A**), 6 h (**B**), and 12 h (**C**), as determined by RT-qPCR. Protein expression levels of inflammatory cytokines in IECs treated with LPS for 3 h (**D**), 6 h (**E**), and 12 h (**F**), as measured by ELISA. * *p* < 0.05 compared to the 0 μg/mL LPS. Data are presented as mean ± SD (*n* = 3 independent IEC isolations). Statistical significance was determined by one-way ANOVA with Tukey’s post hoc test for multiple comparisons.

As shown in Figure 3A–C, LPS treatment modulated the mRNA expression of inflammatory cytokines in a time-dependent and concentration-dependent manner. At 3 h, 1 μg/mL LPS significantly increased the mRNA level of *TNF-α* (*p* < 0.05), while treatment with 5 μg/mL LPS significantly elevated the mRNA levels of *IL-6*, *IL-8*, *IL-1β*, and *TNF-α* and significantly reduced that of *IL-10* (*p* < 0.05). At 6 h, 1 μg/mL LPS significantly increased the mRNA levels of *IL-6*, *IL-8*, and *IL-1β* (*p* < 0.05), and 5 μg/mL LPS significantly enhanced the mRNA levels of *IL-6*, *IL-8*, *IL-1β*, and *TNF-α*, while significantly decreasing that of *IL-10* mRNA (*p* < 0.05). At 12 h, treatment with both 1 and 5 μg/mL LPS significantly increased the mRNA levels of *IL-6*, *IL-1β*, and *TNF-α* while significantly reducing that of *IL-10* (*p* < 0.05).

As illustrated in Figure 3D,F, LPS exposure induced significant alterations in the secretion of inflammatory cytokines in a time-dependent and concentration-dependent manner. At 3 h, 1 μg/mL LPS significantly increased the expression level of IL-8 (*p* < 0.05). In contrast, 5 μg/mL LPS significantly elevated the expression levels of IL-6, IL-8, IL-1β, and TNF-α, and reduced considerably IL-10 (*p* < 0.05). At 6 h, 1 μg/mL LPS significantly increased the expression levels of IL-6 and IL-1β (*p* < 0.05), the 5 μg/mL LPS significantly enhanced the expression levels of IL-6, IL-8, IL-1β, and TNF-α, significantly decreased that of IL-10 (*p* < 0.05). After 12 h, 5 μg/mL LPS significantly increased the secretion of IL-8, IL-10, and IL-1β (*p* < 0.05). Based on the aforementioned experimental data, treatment with 5 μg/mL LPS for 6 h can induce significant expression of pro-inflammatory factors without causing cell death, thereby establishing a stable and sensitive IECs inflammation model for subsequent mechanistic investigations.

### 3.4. Cytotoxicity of F11.1G on IECs

**Figure 4:**
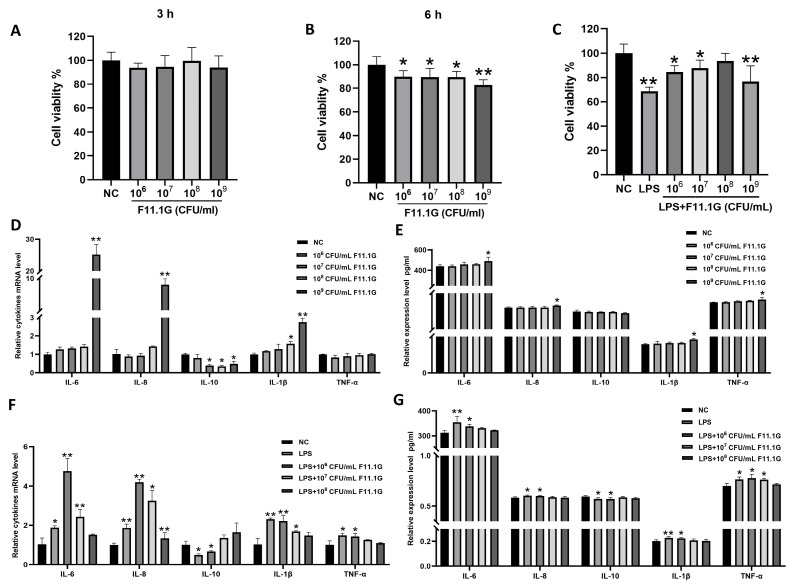
Effects of F11.1G on IECs. Viability of IECs after treatmented with F11.1G for 3 h (**A**) and 6 h (**B**). Viability of IECs following F11.1G intervention for 6 h after LPS-induced inflammatory injury (**C**). mRNA levels (**D**) and protein expression levels (**E**) of inflammatory cytokines in IECs after F11.1G treatment. mRNA levels (**F**) and protein expression levels (**G**) of inflammatory cytokines in IECs following F11.1G intervention after LPS-induced inflammatory injury. * *p* < 0.05, ** *p* < 0.01 compared to the NC group. Data are presented as mean ± SD (*n* = 3 independent IEC isolations). Statistical significance was determined by one-way ANOVA with Tukey’s post hoc test for multiple comparisons.

The cytotoxic effect of F11.1G on IECs was evaluated using the CCK-8 assay. As shown in Figure 4A,B, a significant reduction in cell viability was observed at 6 h of treatment with F11.1G compared to NC group (*p* < 0.05). In contrast, no significant difference in cell viability was detected at 3 h (*p* > 0.05). Furthermore, the impact of F11.1G on the expression of inflammatory cytokines for 3 h was assessed. According to Figure 4D, concentrations ranging from 10^6^ to 10^8^ CFU/mL F11.1G did not significantly affect the mRNA levels of *IL-6*, *IL-8*, *IL-1β*, or *TNF-α* (*p* > 0.05). However, a high concentration of F11.1G (10^9^ CFU/mL) significantly increased the mRNA levels of *IL-6*, *IL-8*, and *IL-1β* (*p* < 0.01). Additionally, concentrations ranging from 10^7^ to 10^9^ CFU/mL F11.1G significantly reduced *IL-10* mRNA level (*p* < 0.05). Consistently, at the protein level, Figure 4E confirms that 10^9^ CFU/mL F11.1G significantly increased the secretion of IL-6, IL-8, IL-1β, and TNF-α (*p* < 0.05).

### 3.5. The Reparative Effect of F11.1G on LPS-Induced Inflammatory Injury in IECs

The reparative potential of F11.1G was evaluated by applying it to IECs for 3 h following LPS-induced injury. CCK-8 results are presented in Figure 4C. Compared with the NC group, cell viability was significantly reduced (*p* < 0.05) following treatment of injured cells with 10^6^, 10^7^, and 10^9^ CFU/mL F11.1G, except the 10^8^ CFU/mL F11.1G group. As shown in Figure 4F, compared with the NC group, treatment of damaged cells with 10^6^ and 10^7^ CFU/mL F11.1G significantly increased mRNA expression levels of *IL-6*, *IL-8*, and *IL-1β* (*p* < 0.05). 10^6^ CFU/mL F11.1G significantly increased *TNF-α* mRNA expression in injured cells (*p* < 0.05), while markedly decreasing *IL-10* mRNA expression (*p* < 0.05). Figure 4G confirms that 10^6^ CFU/mL F11.1G significantly increased the production of IL-6, IL-8, IL-1β, and TNF-α expression levels (*p* < 0.05), while markedly decreasing IL-10 mRNA expression (*p* < 0.05). 10^7^ CFU/mL F11.1G significantly increased the production of TNF-α expression levels. Therefore, we selected the 10^8^ CFU/mL F11.1G treated for 3 h to conduct the LPS-induced IEC inflammatory damage repair experiment.

### 3.6. Effects of LPS-Induced Damage and F11.1G Repair on Differentially Expressed Genes (DEGs) Based on RNA-seq

**Figure 5:**
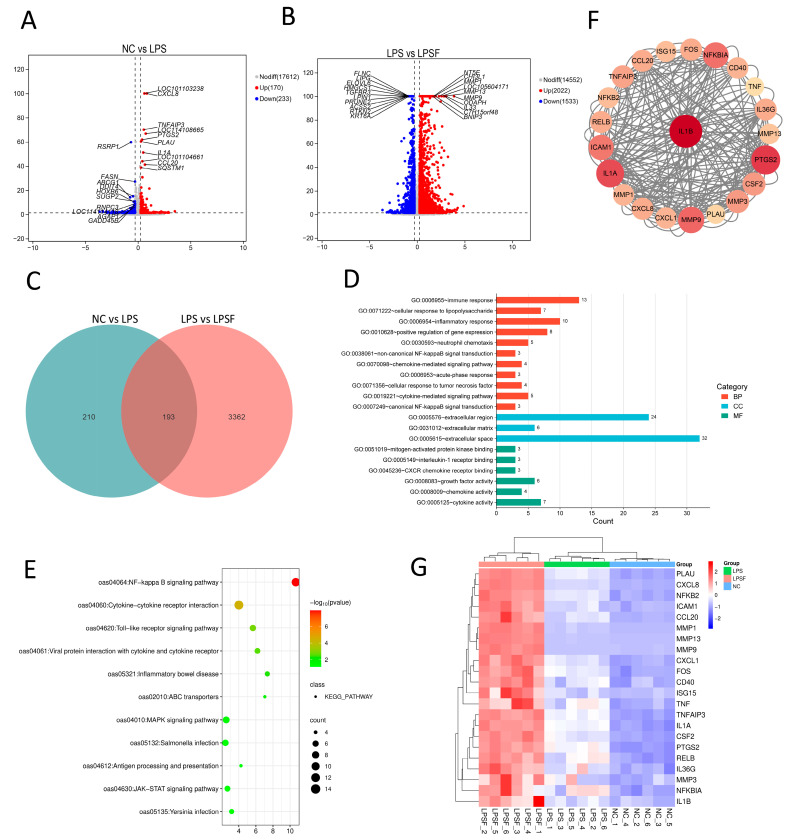
Transcriptomic screening of DEGs. (**A**) Volcano plot of DEGs in NC vs. LPS comparison. (**B**) Volcano plot of DEGs in LPS vs. LPSF comparison. (**C**) Venn diagram of shared DEGs from NC vs. LPS and LPS vs. LPSF comparisons. (**D**) GO functional annotation of DEGs from NC vs. LPS and LPS vs. LPSF comparisons. (**E**) KEGG pathway enrichment analysis of DEGs from NC vs. LPS and LPS vs. LPSF comparisons. (**F**) PPI network of DEGs from NC vs. LPS and LPS vs. LPSF comparisons. (**G**) Heatmap of DEGs identified by PPI network analysis. *n* = 6 independent IEC isolations.

**Figure 6:**
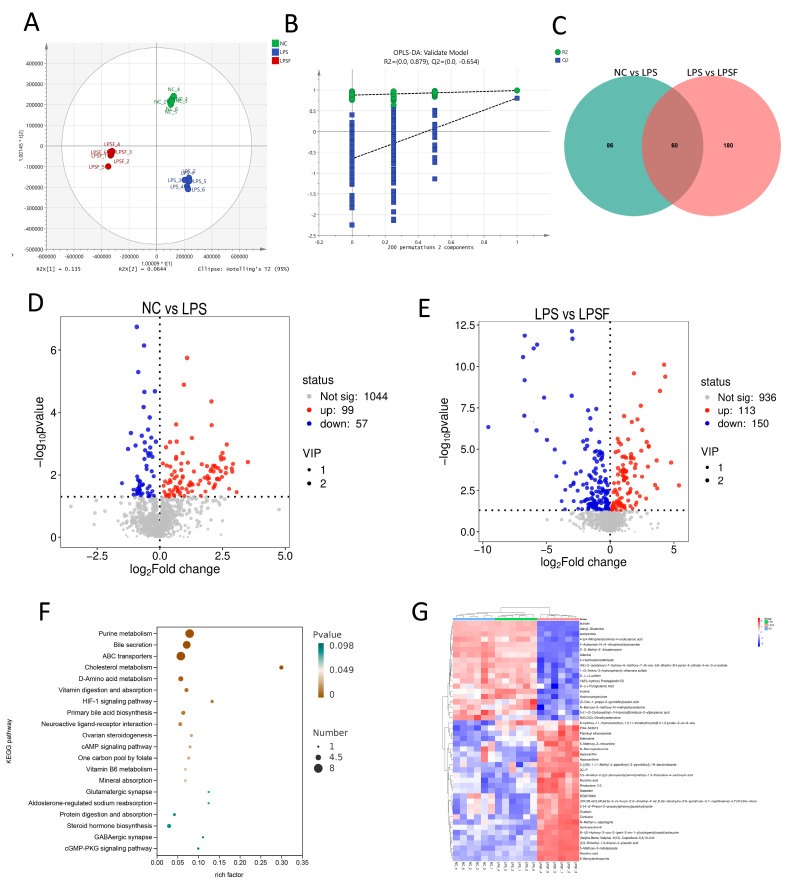
Metabolomics screening of DAMs. (**A**) OPLS-DA plot of DAMs. (**B**) Permutation test plot of DAMs. (**C**) Venn diagram of DAMs in NC vs. LPS and LPS vs. LPSF. (**D**) Volcano plot of DAMs in NC vs. LPS. (**E**) Volcano plot of LPS vs. LPSF. (**F**) KEGG functional annotation of DAMs in NC vs. LPS and LPS vs. LPSF. (**G**) Heatmap showing the abundance patterns of key DAMs across experimental groups. *n* = 6 independent IEC isolations.

DESeq2 was employed for inter-group comparisons, with screening criteria set at |log(FoldChange)| > 1.2 and adjust *p*-value < 0.05. The transcriptomic data evaluation statistics for IECs are provided in Table S1. The volcano plot features log2(FoldChange) on the *x*-axis, representing the magnitude of gene expression change, and −log10 (*p*-value) on the *y*-axis, indicating statistical significance. The volcano plot revealed that LPS treatment induced substantial significant expression alterations in numerous genes compared to the NC group, with upregulated (170) and downregulated (233) genes constituting substantial proportions (Figure 5A). This indicates that LPS may activate or inhibit specific biological pathways, thereby inducing significant changes in transcriptional levels within cells or tissues. Under F11.1G treatment, a substantial number of genes exhibited significant upregulation (2022) or downregulation (1533) compared to the LPS group (Figure 5B), suggesting F11.1G may exert a degree of regulatory influence on LPS-induced gene expression patterns. A total of 193 DEGs were identified from the NC vs. LPS and LPS vs. LPSF comparisons, potentially associated with F11.1G-mediated inflammatory repair (Figure 5C), detailed information on the DEGs is provided in Table S2. GO enrichment analysis of these DEGs revealed predominant enrichment in response to lipopolysaccharide, inflammatory response, non-canonical NF-kappa B signal (NF-κB) transduction, canonical NF-κB signal transduction, and mitogen-activated protein kinase binding (Figure 5D). KEGG (Kyoto Encyclopedia of Genes and Genomes) analysis revealed that DEGs were predominantly enriched in pathways including NF-κB signaling pathway, toll-like receptor signaling pathway, inflammatory bowel disease, MAPK signaling pathway, and JAK–STAT signaling pathway (Figure 6E). The NF-κB and MAPK pathways may represent the signaling routes through which F11.1G mitigates LPS-induced injury, thereby exerting its anti-inflammatory and reparative effects.

PPI network analysis was constructed using the 193 shared DEGs. The top 22 key genes, identified based on degree centrality, exhibited pronounced hub characteristics within the network. Network visualization revealed a positive correlation between node attributes and their degree scores, with this gradient distribution pattern intuitively reflecting the differential importance of genes in the inflammatory regulatory network. Among them, *IL1B (IL-1β)* (degree = 62), possessing the highest connectivity, was identified as the central hub of the entire network. As depicted in Figure 5F, other pivotal genes *(TNF (TNF-α)*, *CXCL8 (IL-8)*, *RELB*, *FOS*, *TNFAIP3*, *NFKBIA*, *MMP9*, etc.) are closely associated with inflammation-related pathways, particularly the NF-κB and MAPK signaling pathways.

To assess the regulatory effect of F11.1G on the LPS-induced inflammatory response, we performed cluster analysis on the 22 DEGs screened from the PPI network. As shown in Figure 5G, there were apparent differences in gene expression levels among the different treatment groups, and all genes clustered into two main categories. Genes including *PLAU*, *IL-8*, *NFKB2*, *ICAM1*, *CCL20*, *MMP1*, *MMP13*, *MMP9*, *CXCL1*, *FOS*, *CD40*, and *ISG15* clustered together and showed the highest expression levels in the LPS group and the lowest in the LPSF group. The remaining genes clustered into another category, including *TNF*, *TNFAIP3*, *IL1A*, *CSF2*, *PTGS2*, *RELB*, *IL36G*, and *MMP3*, with the highest expression in the LPS group, followed by the LPSF group, and the lowest in the NC group. These results indicate that LPS induced a strong inflammatory response, manifested by the activation of the NF-κB pathway and increased expression of downstream genes, including chemokines, cytokines, and matrix metalloproteinases. F11.1G significantly inhibited the upregulation of LPS-induced inflammatory gene expression, demonstrating its ability to suppress the LPS-induced inflammatory cascade.

### 3.7. Effects of LPS-Induced Injury and F11.1G Repair on Differentially Abundant Metabolites (DAMs) Based on Metabolomics Analysis

Principal component analysis was performed using SIMCA 14.1 software. An OPLS-DA supervised model (R^2^X = 0.89, R^2^Y = 0.992, Q^2^ = 0.853) was constructed using DAMs from the NC, LPS, and LPSF groups to analyze the samples. Results indicated that the identified DAMs exhibited intra-group clustering and inter-group separation (Figure 6A). This indicates differences in DAMs between groups. Subsequently, the supervised model was validated using 200 permutations. Results showed that as the number of retained permutations decreased, the R^2^ and Q^2^ values of the random model gradually diminished, confirming the absence of overfitting (Figure 6B), demonstrating that the generated model has good fitting and predictive ability, and the established model is reliable and can be used for DAMs screening. *p* < 0.05, fold change (FC) ≥ 1, and VIP ≥ 1 were used as screening criteria. Compared with the NC group, the LPS group significantly upregulated 99 metabolites and downregulated 57 metabolites (Figure 6D). Under F11.1G treatment conditions, compared with the LPS group, 113 metabolites were significantly upregulated, and 150 metabolites were significantly downregulated (Figure 6E). A total of 60 DAMs screened from NC vs. LPS and LPS vs. LPSF may be related to LPS-induced inflammatory injury in IECs and F11.1G-mediated inflammatory repair (Figure 6C), detailed information on the DAMs is provided in Table S3. KEGG analysis results showed that DAMs were mainly enriched in Purine metabolism, Bile secretion, ABC transporters, HIF-1 signaling pathway, cAMP signaling pathway, and other pathways (Figure 6F).

The 60 DAMs were further filtered using criteria of FDR < 0.01, fold change (FC) > 1, and VIP > 1, resulting in 46 high-confidence DAMs. Subsequently, a heatmap analysis of the expression levels of these DAMs was performed (Figure 6G). Apparent differences in metabolite abundance were observed among the different treatment groups, and all metabolites clustered into two main clusters. The upper metabolite cluster (purine metabolites such as Adenine, Inosine, Adenosine, and Hypoxanthine, etc.) showed the highest abundance in the LPS group and the lowest in the LPSF group. In contrast, the lower metabolite cluster (containing metabolites such as PEA and Niacin) exhibited the opposite trend, with the highest abundance in the LPSF group and the lowest in the LPS group. The differential distribution of these metabolites across treatment groups indicates that LPS stimulation induced significant metabolic disturbances. At the same time, F11.1G intervention effectively regulated the levels of these metabolites and partially restored normal metabolic status. The accumulation of purine metabolites in the LPS group suggests energy metabolism dysregulation and activation of purinergic signaling during inflammation, whereas the reversal of these metabolite levels after F11.1G treatment indicates that its anti-inflammatory effect may be mediated through the regulation of purine metabolism.

### 3.8. Correlation Analysis Between DEGs and DAMs

**Figure 7:**
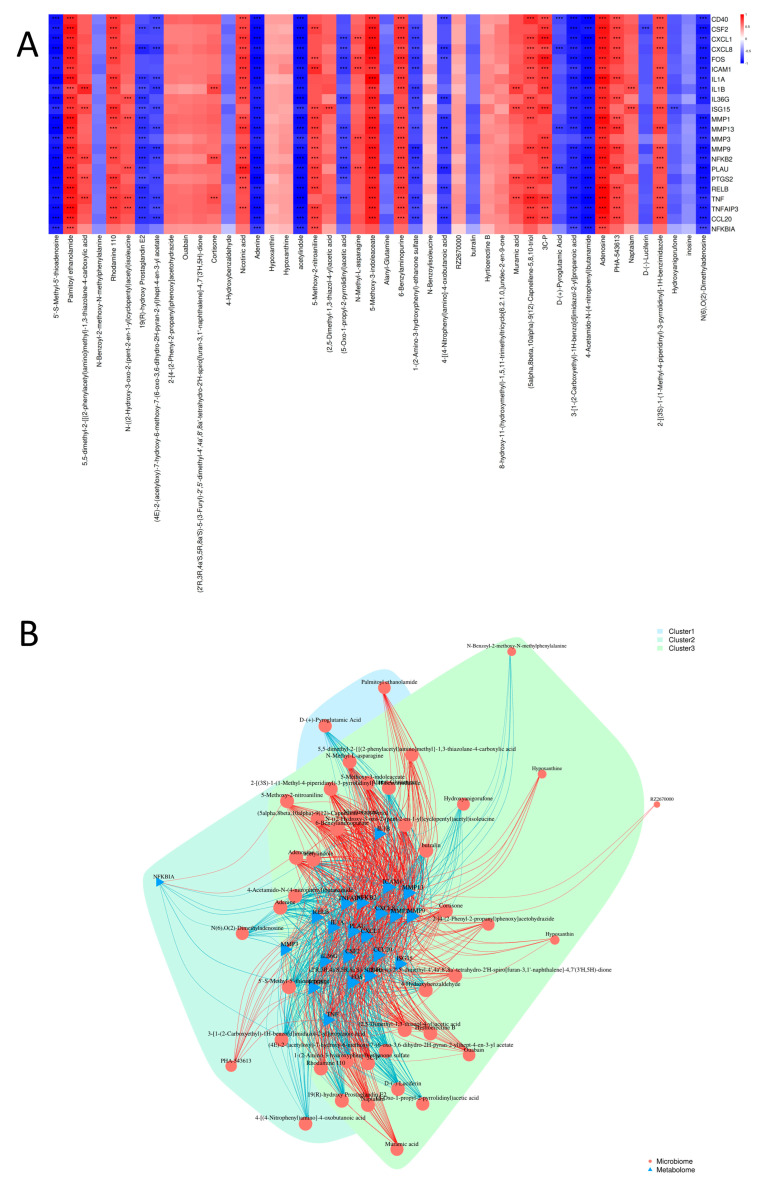
Correlation analysis between DEGs and DAMs. (**A**) Heatmap of Spearman correlations between DEGs and DAMs. (**B**) Interaction network between DEGs and DAMs. Note: Triangles represent DEGs from transcriptomic analysis; circles represent DAMs from metabolomic analysis; different colors indicate different clusters; edge colors represent correlation values (red for positive, blue for negative correlations), with color intensity reflecting the strength of correlation. *** *p* < 0.001.

Spearman correlation analysis between DEGs and DAMs was performed based on |R| > 0.07 and FDR < 0.01, with the results visualized in a heatmap (Figure 7A). *TNF (TNF-α)* showed significantly positive correlations with Adenosine, Palmitoyl ethanolamide (PEA), 5-Methoxy-3-indoleaceate, (5alpha,8beta,10alpha)-9(12)-Capnellene-5,8,10-triol, and 3C-P (*p* < 0.01), while it exhibited significantly negative correlations with acetylindole, 4-Acetamido-N-(4-nitrophenyl)butanamide, Adenine, 3-[1-(2-Carboxyethyl)-1H-benzo[d]imidazol-2-yl]propanoic acid, 5′-S-Methyl-5′-thioadenosine, and N(6),O(2)-Dimethyladenosine (*p* < 0.01). *IL1A* was significantly positively correlated with 5-Methoxy-3-indoleaceate, Adenosine, PEA, and Rhodamine 110 (*p* < 0.01), and significantly negatively correlated with 5′-S-Methyl-5′-thioadenosine, 4-Acetamido-N-(4-nitrophenyl)butanamide, Adenine, acetylindole, 3-[1-(2-Carboxyethyl)-1H-benzo[d]imidazol-2-yl]propanoic acid, and N(6),O(2)-Dimethyladenosine (*p* < 0.01). *IL-8* showed significantly positive correlations with 5-Methoxy-3-indoleaceate, Adenosine, and PEA (*p* < 0.01), and significantly negative correlations with 5′-S-Methyl-5′-thioadenosine, acetylindole, Adenine, 4-Acetamido-N-(4-nitrophenyl)butanamide, N(6),O(2)-Dimethyladenosine, 3-[1-(2-Carboxyethyl)-1H-benzo[d]imidazol-2-yl]propanoic acid, and 19(R)-hydroxy Prostaglandin E2 (*p* < 0.01). *RELB* was significantly positively correlated with Adenosine, PEA, and 5-Methoxy-3-indoleaceate (*p* < 0.01), and significantly negatively correlated with acetylindole, 5′-S-Methyl-5′-thioadenosine, 4-Acetamido-N-(4-nitrophenyl)butanamide, N(6),O(2)-Dimethyladenosine, Adenine, 3-[1-(2-Carboxyethyl)-1H-benzo[d]imidazol-2-yl]propanoic acid, and 19(R)-hydroxy Prostaglandin E2 (*p* < 0.01). *FOS* showed significantly positive correlations with 5-Methoxy-3-indoleaceate, 3C-P, Adenosine, PEA, and 6-Benzylaminopurine (*p* < 0.01), and demonstrated significantly positive correlations with Adenine, 4-Acetamido-N-(4-nitrophenyl)butanamide, acetylindole, N(6),O(2)-Dimethyladenosine, 5′-S-Methyl-5′-thioadenosine, and 3-[1-(2-Carboxyethyl)-1H-benzo[d]imidazol-2-yl]propanoic acid (*p* < 0.01). *NFKB2* was significantly positively correlated with Adenosine, 5-Methoxy-3-indoleaceate, and PEA (*p* < 0.01), and significantly negatively correlated with acetylindole, Adenine, 5′-S-Methyl-5′-thioadenosine, 4-Acetamido-N-(4-nitrophenyl)butanamide, N(6),O(2)-Dimethyladenosine, 3-[1-(2-Carboxyethyl)-1H-benzo[d]imidazol-2-yl]propanoic acid, and 19(R)-hydroxy Prostaglandin E2 (*p* < 0.01). *MMP9* showed significantly positive correlations with 5-Methoxy-3-indoleaceate, Adenosine, and PEA (*p* < 0.01), while exhibiting significantly negative correlations with 5′-S-Methyl-5′-thioadenosine, N(6),O(2)-Dimethyladenosine, Adenine, acetylindole, 4-Acetamido-N-(4-nitrophenyl)butanamide, 3-[1-(2-Carboxyethyl)-1H-benzo[d]imidazol-2-yl]propanoic acid, 1-(2-Amino-3-hydroxyphenyl)-ethanone sulfate, and 19(R)-hydroxy Prostaglandin E2 (*p* < 0.01).

To further investigate the regulatory relationships between DEGs and DAMs, an interaction network was constructed based on 22 genes and 46 metabolites with significant correlations (|R| > 0.7, FDR < 0.01) (Figure 7B). The network consisted of three clusters, each with 29, 26, and 10 nodes, respectively. Analysis revealed that genes and metabolites do not function independently but form tightly connected regulatory modules, uncovering potential molecular mechanisms underlying the LPS-induced inflammatory response and F11.1G-mediated repair.

### 3.9. Validation of Pathway Activity Inhibition by F11.1G in LPS-Induced Inflammatory Injury of IECs

**Figure 8:**
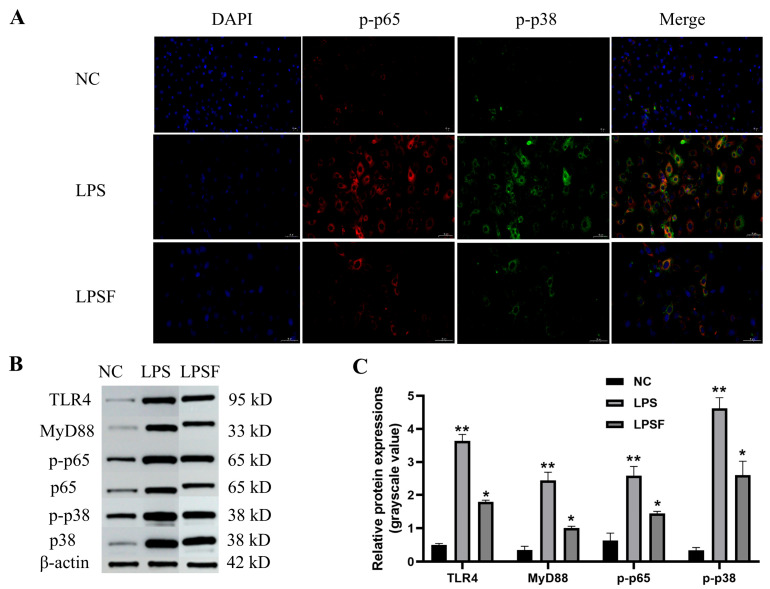
Detection of signaling pathway-related proteins. (**A**) Nuclear translocation of p-p65 and p-p38 detected by IF staining (200× magnification). (**B**) Protein expression levels of signaling pathway-related molecules determined by WB. (**C**) Grayscale quantification of WB results for signaling pathway-related proteins. * *p* < 0.05, ** *p* < 0.01 compared to the NC group. Data are presented as mean ± SD (*n* = 3 independent IEC isolations). Statistical significance was determined by one-way ANOVA with Tukey’s post hoc test for multiple comparisons.

Transcriptomic analysis demonstrated that DEGs were primarily enriched in the NF-κB and MAPK signaling pathways; we further validated the expression of key proteins in these pathways. As shown in Figure 8A, IF analysis revealed that LPS stimulation significantly enhanced the fluorescence intensity of p-p65 and p-p38 proteins in IECs, with prominent nuclear localization. In contrast, F11.1G treatment markedly suppressed the nuclear expression of p-p65 and p-p38, leading to their redistribution into the cytoplasm and a gradual reduction in fluorescence intensity. WB results demonstrated that LPS treatment significantly upregulated the expression of TLR4, MyD88, p-p65, and p-p38 (*p* < 0.01), indicating activation of both the NF-κB and MAPK pathways (Figure 8B,C). However, F11.1G intervention significantly downregulated the expression of TLR4, MyD88, p-p65, and p-p38 compared to the LPS group (*p* < 0.05), while remaining significantly higher than the NC group (*p* < 0.05). These findings confirm that F11.1G alleviates LPS-induced inflammatory injury in IECs by inhibiting the TLR4/MyD88/NF-κB/MAPK pathway, which is consistent with the pathway predictions from transcriptomic analysis.

## 4. Discussion

IECs form the first line of defense of the intestinal barrier through the secretion of mucins, antimicrobial peptides, and inflammatory factors. LPS, a key component of Gram-negative bacterial cell walls, disrupts intestinal epithelial integrity upon release during bacterial lysis, and can trigger systemic inflammatory responses [24,25]. In vitro studies demonstrated that 1–20 μg/mL of LPS significantly reduced the viability of IECs at 6 h and 12 h. Treatment with 5 μg/mL LPS for 6 h can establish an optimal inflammation model, characterized by increased expression of *IL-6*, *IL-8*, *IL-1β*, and *TNF-α*, while *IL-10* levels decrease. Building on previous findings that intracellular metabolites from *Lactobacillus* KFCC11389P suppress pro-inflammatory cytokines in macrophages, while live or heat-killed bacteria show no such effects [26], we investigated F11.1G. Preliminary tests revealed that both live and heat-killed F11.1G induced cytotoxicity or inflammation in IECs. Among tested concentrations, treatment with 10^8^ CFU/mL F11.1G for 3 h effectively reduced inflammatory factor expression without cytotoxicity, unlike ineffective 10^6^ CFU/mL or pro-inflammatory 10^9^ CFU/mL treatments. These findings are consistent with reports indicating that *Lactobacillus curvatus* WiKim38 alleviates colitis in mice by activating the NF-κB and ERK signaling pathways, leading to increased production of the anti-inflammatory cytokine *IL-10* [27]. Mechanistically, LPS triggers the activation of TLR4 on IECs, leading to the activation of MyD88 and NF-κB pathway. This activation simultaneously induces the production of pro-inflammatory cytokine and suppresses the expression of tight junction protein. The consequent loss of intestinal barrier integrity allowed luminal LPS and pathogens to invade the submucosa, which exacerbates intestinal inflammation and barrier damage [28]. This supports that targeting the TLR4/MyD88/NF-κB pathway could help prevent the inflammatory injury caused by LPS.

LPS is a key component of Gram-negative bacterial cell walls, binds to TLR4 receptors on the surfaces of IECs, and activates inflammatory signaling pathways such as NF-κB and MAPK. The activation of these pathways promotes the secretion of pro-inflammatory factors such as *TNF-α* and *IL-6* [29,30]. To investigate the effect of F11.1G on the LPS-induced inflammatory model in IECs, transcriptomic analysis was performed. The obtained DEGs were enriched in inflammation- and immune-related pathways, including the MAPK signaling pathway and the NF-κB signaling pathway. The NF-κB family regulates multiple signaling pathways within the immune system, enabling cells to react to various pro-inflammatory triggers. Activation of the NF-κB pathway can occur due to factors such as bacterial toxins, viruses, ultraviolet radiation, and oxidative stress [31]. The MAPK signaling pathway plays a crucial role in cell survival, migration, and autophagy, serving as a key regulatory target that connects cell surface receptors to intracellular processes [32]. Studies have shown that exopolysaccharides (EPS) derived from lactic acid bacteria can stimulate the host’s immunomodulatory responses through the intestinal mucosal immune system. This immunoregulatory function commences with the recognition of EPS by pattern recognition receptors on the surface of IECs or immune cells, subsequently activating pathways such as NF-κB and MAPK [33]. Notably, the specific immunological effects of this pathway activation vary depending on the structural characteristics of the EPS and the cellular environment. For instance, the EPS secreted by *Lactobacillus pantheris* TCP102 activates macrophages via the aforementioned pathways, promoting the expression and release of pro-inflammatory mediators such as *TNF-α*, *IL-6*, and *nitric oxide (NO)*. This response contributes to its immunostimulatory and potential antitumor effects [34]. Conversely, *Limosilactobacillus mucosae* CCFM1273 EPS decreased LPS level, which in turn inhibited the TLR4/NF-κB pathway, alleviating ulcerative colitis in mice [35]. These findings collectively indicate that exogenous stimulus-mediated activation of the NF-κB and MAPK pathways can induce either immune stimulation or immunosuppression, thereby exerting regulatory effects on mucosal immune homeostasis. Transcriptomic analysis indicated that DEGs were enriched in the NF-κB and MAPK signaling pathways, suggesting that F11.1G may exert immunostimulatory effects through intracellular components such as EPS. WB analysis at the protein level demonstrated that LPS stimulation increased the protein expression of TLR4, MyD88, p-p65, and p-p38 in IECs, as well as enhanced the fluorescence intensity of p-p65 and p-p38, thereby activating the NF-κB and MAPK pathways. In this study, after F11.1G treatment, the fluorescence intensity of p-p65 and p-p38 in IECs decreased. The WB and IF results in this study were consistent, suggesting that F11.1G may reduce the expression of TLR4 and MyD88, thereby interrupting the NF-κB and MAPK signaling pathways. This would inhibit the secretion of inflammatory factors such as *IL-6*, *IL-8*, *IL-1β*, and *TNF-α*, and reduce LPS-induced inflammatory damage in IECs.

Studies have shown that pre-treatment with supernatants from lactic acid bacteria *Leuconostoc mesenteroides*, *Latilactobacillus curvatus*, and *Lactiplantibacillus plantarum* significantly reduces the activation of phosphorylated p38 protein in RAW264.7 macrophages and decreases the expression of inflammatory factors such as *TNF-α*, *IL-1β*, and *IL-6* [36]. The anti-inflammatory activity is mediated by the p38 signaling pathway. Karthikeyan et al. [37] also reported that solvent components extracted from the ethyl acetate soluble supernatant of *Lactobacillus casei* inhibit the phosphorylation of NF-κB and ERK1/2 in LPS-induced peripheral blood mononuclear cells and HT-29, downregulate *TNF-α* and *IL-6* genes, upregulate *IL-10*, and exert anti-inflammatory potential, which is consistent with the results of this study. Meanwhile, we found that after treatment with ultrasonically disrupted F11.1G on injured cells, the phosphorylation levels of MAPK and NF-κB were higher than NC group but lower than LPS group, confirming that the protein phosphorylation of this pathway was effectively inhibited compared to the LPS group. This indicates that at the protein level, substances in F11.1G, such as exopolysaccharides, may have the ability to awaken cellular immune functions while potentially inhibiting LPS-induced excessive phosphorylation, which aligns with the findings of Xu et al. (2022) [33]. This further confirms that substances in F11.1G may inhibit LPS-induced cellular inflammation. The multi-level regulation, from gene expression to protein activity, may be the core mechanism by which F11.1G effectively alleviates excessive inflammation without compromising immune defense capabilities.

Based on PPI results, this study screened core genes closely related to NF-κB and MAPK signaling pathways, including *IL-1β*, *TNF-α*, *IL-8*, *RELB*, *FOS*, *TNFAIP3*, *NFKBIA*, and *MMP9*, as subsequent validation targets. *IL-1β* induces the activation of NF-κB and MAPK signaling pathways and participates in regulating the expression of multiple inflammation-related genes [38]. In THP-1 macrophages, *TNF-α*, expression is upregulated after LPS stimulation, and NF-κB activity regulates TNF-induced expression in cells [39], NF-κB signal inhibition attenuates LPS-induced *TNF-α* expression [40]; in LPS-induced endotoxemia mice model, mice initiate innate immune responses through NF-κB subunit nuclear translocation and *TNF-α* gene induction [41]. *CXCL8*, also known as *IL-8*, is a CXC chemokine with pro-inflammatory properties [42]. *IL-8* promotes inflammatory responses, angiogenesis, cell division, and proliferation by binding to two receptors: *CXC chemokine receptor 1* (*CXCR1*) and *CXC chemokine receptor 2* (*CXCR2*) [43]. NF-κB family transcription factors consist of five members: p50, p52, p65 (RELA), C-REL and RELB, encoded by *NFKB1*, *NFKB2*, *RELA*, *REL* and *RELB*, respectively, related to NF-κB transcriptional activation regulatory functions [44], LPS also induces *p50 (NF-κB 1)*, *p65 (RELA)* and *MyD88* mRNA levels in THP-1 macrophages [45]. *FOS*, as a key early immediate response gene and a core member of the AP-1 transcription factor family, is precisely regulated by the MAPK signaling pathway through the activation of *FOS* gene transcription and the phosphorylation of expressed proteins, thereby achieving comprehensive control of *FOS* function and exhibiting rapid, transient expression characteristics [46]. *TNFAIP3*, also known as zinc finger protein A20, is a rare enzyme that possesses both ubiquitination and deubiquitination activities, and can inhibit the inflammatory NF-κB signaling pathway [47]. *MMP9*, also known as gelatinase B, is a secreted protease that accelerates the maturation of cytokines such as *IL-1β* and *TNF-α*, indicating the activation of inflammatory responses and tissue damage. MAPK signaling pathways, including ERK1/2, JNK1/2, and p38, participate in *MMP-9* production [48]. Transcriptome data and RT-qPCR verification both demonstrated that LPS stimulation significantly upregulated pro-inflammatory gene *IL-1β* and chemokine gene *IL-8* mRNA expression (Figure 4). To confirm whether this transcriptional change ultimately led to functional protein secretion, ELISA results showed IL-1β and IL-8 protein expressions in cell supernatant were significantly increased, and after F11.1G treatment, both inflammatory factors’ secretion was effectively inhibited (Figure 5). Protein level demonstrates that F11.1G alleviates LPS-stimulated IECs inflammatory injury, which relates to the NF-κB pathway and the MAPK pathway, proving F11.1G coordinately exerts anti-inflammatory effects at both gene transcription and protein secretion levels. The DEGs heatmap revealed elevated expression levels of *IL-8*, *IL-1*, *TNF-α*, and *IL1A* in the LPS group, while their expression decreased in the LPSF group. This further confirms that LPS induced cellular inflammation, and F11.1G intervention alleviated the occurrence of cellular inflammation. NF-κB pathway-related genes, such as *NFKB2* and *RELB*, were upregulated in the LPS group and downregulated in the LPSF group, indicating that LPS triggered cellular inflammation. F11.1G may exert its anti-inflammatory effects by regulating the NF-κB signaling pathway.

Clinical diseases typically involve interactions between multiple genes, pathways, and environmental factors, making it difficult for a single-omics modality to provide a comprehensive analysis. Multi-omics integration is emerging as a core strategy for precise diagnosis and classification [49,50]. Non-targeted metabolomics identified 46 DAMs, which were mainly enriched in pathways including Purine metabolism, Bile secretion, ABC transporters, HIF-1 signaling pathway, and cAMP signaling pathway. Among them, Adenine, Inosine, Adenosine, and Hypoxanthine are associated with the purine metabolism pathway. Purine metabolism is linked to amino acid metabolism through the purine nucleotide cycle [51] and plays a key role in metabolic regulation, coenzyme generation, and energy supply. Yuan et al. (2023) [52] suggested that nervonic acid may modulate purine metabolism to protect mice from colitis infection. PEA is an endogenous lipid mediator with potent anti-inflammatory and analgesic functions. PEA can be hydrolyzed by the lysosomal enzyme N-acylethanolamine acid amidase (NAAA), which is highly expressed in macrophages and other immune cells. Pharmacological inhibition of NAAA activity is a potential strategy for treating inflammation-related diseases [53]. Nicotinic acid (vitamin B3, NA) is a key mediator of neural development and survival in the central nervous system. NA can inhibit LPS-induced activation of the NLRP3 inflammasome and the release of pro-inflammatory factors such as IL-1β through the SIRT1/PARP-1/NLRP3 cascade [54]. Glucocorticoids can be classified into natural hormones, including cortisone and cortisol, and synthetic hormones, including dexamethasone, prednisone, and methylprednisolone [55]. Studies have shown that cortisol suppresses the NF-κB and MAPK signaling pathways as well as LPS-TLR4-mediated inflammatory responses, thereby regulating excessive immune activation while promoting inflammatory repair [56]. During the acute phase, activation of the hypothalamic–pituitary–adrenal (HPA) axis promotes the release of endogenous glucocorticoids, forming a self-regulatory mechanism. However, this feedback mechanism fails in chronic inflammation, and exogenous glucocorticoids can help restore the balance between anti-inflammatory responses and tissue repair [57]. The metabolomics heatmap revealed that LPS challenge induced systemic metabolic dysregulation, primarily involving the abnormal activation of purinergic signaling, dysregulation of the endocannabinoid system, and an imbalance in the neuro-immune–endocrine network. After F11.1G treatment, the levels of these metabolites were restored, indicating its potential multi-target synergistic anti-inflammatory effects: firstly, purine metabolic balance is regulated, which correlates with the Purine metabolism pathway predicted by metabolomics. Secondly, by activating the endocannabinoid system (e.g., PEA) to enhance the body’s self-protective mechanisms. Gut microbiota activates the ileal nuclear receptor farnesoid X receptor (FXR) to inhibit secondary bile acid metabolism and bile acid production in the liver, thereby controlling hepatic inflammation. This receptor overlaps with core receptors of the endocannabinoid system, collectively forming a synergistic network regulating liver inflammation and metabolism [58]. Studies have shown that the ABCG2 member of the ABC transporter family is directly involved in the transport of endocannabinoids (e.g., Anandamide), indicating improved endocannabinoid homeostasis and signaling [59]. Therefore, bile secretion is related to the endocannabinoid system. Thirdly, by modulating HPA axis hormone levels and neuro-immune interactions, F11.1G collectively reverses metabolic imbalance and restores internal homeostasis, thereby alleviating inflammatory injury. Bousquet et al. (2001) [60] found that the cAMP signaling pathway, in synergy with cytokines, regulates key factors such as *cytokine signaling-3 (SOCS-3)*, directly participating in the fine-tuning of HPA axis activity, as well as subsequent glucocorticoid release and anti-inflammatory signaling. The HIF-1 signaling pathway and NF-κB pathway act synergistically in inflammation, with NF-κB driving HIF-1α transcription, together forming a pro-inflammatory cascade signaling pathway. Blocking this pathway can effectively inhibit inflammation [61]. In summary, the identified DAMs correspond with their KEGG-predicted pathway results.

Spearman correlation analysis between DEGs and DAMs revealed a metabolomic regulatory network closely associated with inflammatory responses. We found that the screened core pro-inflammatory genes (*TNF-α*, *IL-1α*, *IL-8*, *MMP9*, and the NF-κB pathway components *RELB* and *NFKB2*) all showed significantly positive correlations with Adenosine, PEA, and 5-methoxy-3-indoleacetate. The secretion of inflammatory mediators such as *TNF-α* directly impacts intestinal epithelial tissue, leading to disruption of the epithelial barrier and inducing epithelial cell apoptosis [62]. Research indicates that *MMP-9* can induce expression of the NF-κB target gene *IL-8* [63], resulting in increased permeability of TJ within the intestinal epithelium and thereby contributing to the development of intestinal inflammation in inflammatory bowel diseases [64]. In colorectal cancer, *RELB* promotes cell proliferation by directly transcribing proteins such as Bcl-3, and is associated with a poor patient prognosis [65], serving as an early warning marker in acute inflammation models. F11.1G effectively suppresses excessive activation of this pathway through its metabolites (e.g., Adenosine), thereby exhibiting acute anti-inflammatory efficacy. Adenosine inhibits NF-κB activation and reduces downstream inflammatory factor *TNF-α* production, which may represent a key mechanism for its cardioprotective effects [66]. In this study, its positive correlation with pro-inflammatory genes precisely reflects the activation of this endogenous protective pathway under LPS challenge. Similarly, PEA, as an endogenous lipid mediator, has been demonstrated to modulate PPAR-α to inhibit NF-κB activation, thereby promoting microglial polarization toward an anti-inflammatory phenotype and alleviating neuroinflammation [67]. Its coordinated upregulation with pro-inflammatory genes suggests that F11.1G may enhance this endogenous negative feedback mechanism. Meanwhile, correlation heatmap and network analyses also revealed a more direct pro-inflammatory metabolic signature: pro-inflammatory genes showed significantly negative correlations with purine metabolites (Adenine, 5′-S-Methyl-5′-thioadenosine, N(6),O(2)-Dimethyladenosine, etc.). This suggests that under pro-inflammatory conditions, purine metabolism is significantly suppressed or purine nucleosides are substantially consumed to support the activation and proliferation of immune cells. Previous research has indicated that purine metabolites such as Adenine can serve as therapeutic targets for immune regulation and prevention of inflammatory injury [68]. The purine metabolism pathway enriched by DAMs may represent a typical metabolic signature of cellular immune activation. However, it should be noted that the core mechanism of F11.1G may lies in enhancing cellular responsiveness to it. Consequently, the most direct pharmacological target is not Adenosine itself, but its receptors (A3R, A2BR, A2AR, and A1R). Signaling of these receptors reduces the function of immune effector cells and enhances the expansion and function of tumor-associated immune cells [69]. However, we must recognize that pharmacological regulatory mechanisms such as the purine metabolism pathway mediated by purinergic receptors or the endocannabinoid system involved in PEA signaling still require validation through relevant functional experiments.

In summary, ultrasonicated F11.1G may exert multi-target and multi-level regulatory effects by modulating inflammatory genes and related metabolites, thereby influencing the NF-κB/MAPK signaling pathway, maintaining immune balance, and effectively inhibiting the occurrence of inflammation. This provides a theoretical foundation for its development as a potential postbiotic preparation capable of repairing the intestinal barrier and regulating immune homeostasis.

This study focuses on the wild-type strain *Enterococcus faecalium* F11.1G. whose advantage lies in utilizing intact intracellular metabolites prepared via ultrasonic disruption as novel postbiotic formulations. By integrating multi-omics analyses, including transcriptomics and metabolomics, this study aims to construct a synergistic regulatory network that links metabolite enrichment with key signaling pathways. This provides a mechanistic basis for F11.1G’s restorative potential in LPS-induced intestinal inflammation. However, this study also presents certain limitations: pharmacological regulatory mechanisms identified through metabolite enrichment, such as purinergic receptor-mediated purine metabolism pathways or endocannabinoid system PEA signaling, require validation via relevant functional experiments and further confirmation in vivo. We are currently conducting systematic and in-depth functional validation of the regulatory mechanisms of F11.1G through mouse experiments.

## 5. Conclusions

The F11.1G suppresses secretion of pro-inflammatory factors IL-6, IL-1β and TNF-α, suppresses the activation of NF-κB and MAPK signaling pathways, and regulates key genes including *IL-1β*, *TNF-α*, *IL-8* and *RELB*. Furthermore, it may also exhibit specific associations with purine metabolism pathways and key metabolites, such as Adenosine and PEA, within the endocannabinoid system. Through these synergistic actions on inflammatory initiation and repair, F11.1G alleviates LPS-induced cellular damage, demonstrating its potential as an anti-inflammatory agent for lamb intestinal inflammation treatment.

## Figures and Tables

**Table 1:** qPCR primers.

Gene	Forward	Reverse
*IL-6*	5′-ATAACCACTCCAGCCACACA-3′	5′-TGCGTTCTTTACCCACTCGTT-3′
*IL-8*	5′-TGACTTCCAAGCTGGCTGTTG-3′	5′-ATGGATCTTGCTTCTCAGCTCTC-3′
*IL-10*	5′-TTTCTGCCCTGCGAAAACAAG-3′	5′-CCACTTGCTGGGTTCCTACAC-3′
*IL-1β*	5′-CAGCCGTGCAGTCAGTAAAA-3′	5′-GAAGCTCATGCAGAACACCA-3′
*TNF-α*	5′-CCACGTTGTAGCCGACATC-3′	5′-CCCTGAAGAGGACCTGTGAG-3′
*GAPDH*	5′-TGGTGAAGGTCGGAGTGAAC-3′	5′-GGAAGATGGTGATGGGATTTC-3′

## Data Availability

All data, tables, and figures are original and are available in this article.

## Supplementary Materials

The following supporting information can be downloaded at https://www.mdpi.com/article/10.3390/cells15020103/s1, Table S1: IECs transcriptomic data evaluation statistics (*n* = 6 independent IEC isolations). Table S2: Transcriptomic DEGs (*n* = 6 independent IEC isolations). Selection criteria: |log(FoldChange)| > 1.2 and adjust *p*-value < 0.05. Table S3: Metabolomics DAMs (*n* = 6 independent IEC isolations). Selection criteria: FDR < 0.01, fold change (FC) > 1, and VIP > 1.

## Abbreviations

The following abbreviations are used in this manuscript:

LPS	Lipopolysaccharide
IECs	Intestinal epithelial cells
DEGs	Differentially expressed genes
DAMs	Differentially altered metabolites
ETEC	Enterotoxigenic *Escherichia coli*
*E.coli*	*Escherichia coli*
TJs	Tight junctions
IL-6	Interleukin-6
IL-8	Interleukin-8
IL-10	Interleukin-10
IL-1β	Interleukin-1beta
TNF-α	Tumor necrosis factor-alpha
T3PKS	Type III Polyketide synthases
GAPDH	Glyceraldehyde-3-phosphate dehydrogenase
NRPS/NRPS-like	Non-ribosomal peptide synthetases
IF	Immunofluorescence
ELISA	Enzyme-linked immunosorbent assay
MRS	De Man, Rogosa, and Sharpe
OD	optical density
PBS	phosphate-buffered saline
WB	Western blot
GO	Gene Ontology
KEGG	Kyoto Encyclopedia of Genes and Genomes
PPI	Protein–protein interaction
EPS	Exopolysaccharides
NO	Nitric oxide
PEA	Palmitoyl ethanolamide
HPA	Hypothalamic–pituitary–adrenal
SOCS-3	Cytokine signaling-3
