# Supplementary material for: The Mechanism of Ultrasonic Lysis of Enterococcus faecium F11.1G in Repairing LPS-Induced Inflammatory Damage in IECs via RNA-seq and LC-MS
Source: Cells. 2026 Jan 6;15(2):103. doi: 10.3390/cells15020103 (PMC12839353; doi:10.3390/cells15020103)
Supplement: Supplementary file 1 [file cells-15-00103-s001.zip › cells-4035905-supplementary.pdf]

Supplemental Materials

Table S1. IECs transcriptomic data evaluation statistics (n = 6 independent IEC isolations).

Sample	Raw Reads No	Raw Bases(bp)	Q30(bp)	GC(%)	N(%)	Q20(%)	Q30(%)
NC_1	39605800	5.98E+09	5.74E+09	47.17	0.00329	99.08	96.02
NC_2	38680490	5.84E+09	5.61E+09	47.22	0.003339	99.11	96.05
NC_3	42881890	6.48E+09	6.27E+09	47.25	0.022127	99.22	96.82
NC_4	39218176	5.92E+09	5.69E+09	47.18	0.003307	99.1	96.08
NC_5	41257020	6.23E+09	5.99E+09	47.2	0.00332	99.11	96.12
NC_6	46527838	7.03E+09	6.81E+09	47.13	0.02201	99.23	96.9
LPS_1	42309138	6.39E+09	6.14E+09	46.99	0.00326	99.09	96.04
LPS_2	57946510	8.75E+09	8.47E+09	47.1	0.022108	99.19	96.75
LPS_3	38525072	5.82E+09	5.58E+09	47.08	0.00323	99.09	95.93
LPS_4	58461252	8.83E+09	8.55E+09	47.11	0.022145	99.2	96.83
LPS_5	56284650	8.5E+09	8.23E+09	46.91	0.02213	99.19	96.78
LPS_6	57619912	8.7E+09	8.42E+09	46.89	0.022213	99.19	96.72
LPSF_1	38663900	5.84E+09	5.6E+09	46.85	0.003322	99.04	95.93
LPSF_2	48555852	7.33E+09	7.09E+09	46.89	0.022365	99.2	96.75
LPSF_3	47184034	7.12E+09	6.89E+09	46.96	0.022176	99.17	96.66
LPSF_4	38234586	5.77E+09	5.55E+09	46.96	0.003292	99.12	96.08
LPSF_5	45636518	6.89E+09	6.68E+09	46.81	0.007467	99.32	96.92
LPSF_6	59816760	9.03E+09	8.77E+09	46.79	0.007333	99.35	97.14

Table S2. Transcriptomic DEGs (n = 6 independent IEC isolations). Selection criteria: |log(Fold-Change)| > 1.2 and adjust *P*-value < 0.05.

DEGs	NC_vs_LPS		LPS_vs_LPSF	
	|log2fc|	adjust pvalue	|log2fc|	adjust pvalue
CCL20	0.6659	6.17E-42	1.6108	1E-100
CXCL8	0.8544	1E-100	1.4667	1E-100
HBEGF	0.3472	6.78E-12	1.0668	1E-100
INHBA	0.4311	1.76E-31	1.3913	1E-100
MMP1	0.5506	6.64E-09	2.2977	1E-100
MMP13	0.3042	2.34E-09	2.813	1E-100
MMP9	0.4417	0.001967	3.881	1E-100
PLAU	0.2886	5.67E-61	0.4785	1E-100
PRDM1	0.2817	5.86E-07	1.166	1E-100
TGFBR3	0.2645	1.35E-15	0.937	1E-100
FASN	0.274	6.05E-28	0.5766	6.38E-91
ABCA1	0.516	0.000141	2.11	3.85E-88
SLC16A3	0.3023	7.71E-05	1.201	1.91E-80
TNFAIP3	0.5613	8.12E-71	0.5496	8.23E-77
IL1A	0.5087	5.65E-52	0.4782	1.54E-75
DUSP1	0.2923	7.61E-18	0.5761	3.05E-68
NFKB2	0.2706	7.9E-18	0.4965	3.17E-64
SEMA6D	0.5992	0.03704	2.6062	2.89E-57
VSNL1	0.3365	0.000458	1.355	5.32E-56
STEAP4	0.3731	0.002093	1.467	7.62E-53
NR4A3	0.358	0.02165	1.715	2.44E-51
FAM184B	0.3968	0.000177	1.1965	7.44E-48
SLC6A14	0.4868	6.02E-11	0.8924	1.05E-44
SOD2	0.3235	5.35E-23	0.3998	3.19E-44
CSF2	1.0885	4.23E-22	1.0225	4.15E-41
ABCG1	0.7359	7.89E-15	1.1661	4.51E-39
PTGS2	0.4008	1.48E-62	0.3153	7.52E-39
ICAM1	0.3447	0.00082	1.0422	2.17E-35
CTSL	0.3606	1.78E-15	0.478	9.89E-35

NFKBIA	0.2938	1.63E-09	0.5053	5E-33
DDIT4	0.4563	1.04E-15	0.6415	1.5E-31
TNIP1	0.2704	3.89E-09	0.4829	3.97E-31
CXCL1	0.7383	9.73E-09	1.0843	2.06E-30
HMOX1	0.269	4.05E-14	0.4029	3.36E-30
KIF5C	0.3183	0.005445	1.1104	9.29E-29
CD74	0.3667	0.02073	1.2233	2.12E-26
FOS	0.3209	4.58E-12	0.4672	9.72E-26
ALDH1A3	0.3141	0.01578	1.0026	1.41E-25
TMPRSS2	0.2688	0.000203	0.662	2.26E-25
LIF	0.5347	0.000293	1.1885	4.95E-25
ADAM19	0.2795	4.53E-06	0.6009	4.41E-22
IKBKE	0.4481	8.65E-13	0.499	2.54E-21
EDN2	0.2633	0.001822	0.9256	1.95E-19
PLPP3	0.4465	0.04767	1.4551	4.04E-19
AEBP1	0.4072	0.03766	1.5077	5.14E-19
EDNRB	0.3231	0.02993	1.1578	1.76E-17
PRKG2	0.6563	1.01E-05	0.914	1.04E-16
TMPRSS11D	0.3079	0.01499	0.7484	4.12E-16
TMEM255A	0.3074	0.03586	1.0379	6.61E-16
PTPRU	0.4281	0.0265	1.29	7.82E-15
PTX3	0.8503	1.45E-13	0.6904	8.04E-15
ACP3	0.3323	2.25E-18	0.2821	2.06E-14
RNF125	0.4209	0.02252	1.0083	5.07E-13
FCRL3	0.7745	0.006965	1.3026	6.75E-13
CNTNAP2	0.3788	3.09E-05	0.5837	6.5E-12
GABRR3	0.4148	0.03152	1.0283	6.81E-12
MX2	0.8027	1.4E-06	1.6358	8.56E-12
HS6ST2	0.3258	0.00024	0.7369	1.84E-11
KRT23	0.3088	0.03696	0.8174	5.68E-11
CD40	0.2984	0.004859	0.5777	6.38E-11
FAM180A	0.6788	8.44E-06	1.022	1.14E-10
EGR3	0.5584	0.03105	1.193	1.89E-10
ISG15	0.5615	0.04433	1.8654	3.52E-10
PLPPR3	0.4116	0.002504	0.7578	7.45E-10
TG	0.5691	0.004906	1.1315	2.08E-09
BCAT2	0.3059	0.01622	0.7141	2.14E-09
SUGP2	0.2873	1.65E-11	0.2893	3.32E-09
DAGLA	0.4069	0.003618	0.8801	1.07E-08
ATP12A	0.6111	0.02158	1.1811	1.31E-08
ID3	0.3124	2.96E-06	0.3788	1.66E-08
CHRNA3	0.4371	0.02474	0.995	1.7E-08
IL36G	0.5108	0.000201	0.6272	5.45E-08
MUC20	0.2995	0.008485	0.6717	8.65E-08
AUTS2	0.3054	0.003269	0.4815	9.85E-08
PDGFB	0.3222	1.43E-07	0.3118	1.9E-07
RSAD2	0.5242	0.000152	0.9792	3.48E-07
RELB	0.375	9.49E-07	0.3373	4.53E-07
SMPDL3B	0.7973	0.03227	1.5543	5.02E-07
CCDC85A	0.6162	0.03778	1.2638	1.14E-06
FRMD7	0.6305	0.04035	1.2987	1.55E-06
FOXA1	0.265	0.04551	0.5299	2.84E-06
TMEM116	0.4709	0.03019	0.9743	3.06E-06
MCAM	0.4915	0.03192	1.157	3.72E-06
DENND2D	0.2804	0.02497	0.6853	6.7E-06
IL1B	1.5213	0.04618	1.8901	6.98E-06
ABCC6	0.4023	0.000613	0.4701	7.12E-06
GPRIN1	0.3403	0.04257	0.8048	9.38E-06
TNF	0.5605	3.02E-05	0.5501	1.54E-05
SMYD3	0.3078	0.02178	0.706	2.01E-05
PRSS12	0.3196	0.000289	0.365	2.22E-05
TNFAIP6	0.8025	0.03564	0.8729	2.31E-05
PPP1R3F	0.2749	0.0174	0.4607	2.31E-05

BTBD19	0.3083	0.007165	0.4626	2.9E-05
SLC6A12	0.7136	2.53E-05	0.5367	3.29E-05
HAPLN1	0.8915	0.03365	1.6821	3.97E-05
ZNF446	0.3546	0.006909	0.5368	4.98E-05
CYP2J	0.5852	0.01498	0.9568	5.52E-05
NME3	0.3537	0.004317	0.4693	9.22E-05
ID1	0.5741	2.49E-06	0.5062	9.74E-05
EID2	0.6448	0.03225	1.367	0.000102
CCNB1IP1	0.3775	0.000687	0.5017	0.000103
SLC37A1	0.3371	3.28E-05	0.2972	0.000108
IGFLR1	0.2936	0.03305	0.6338	0.000112
ADAMTS20	2.854	0.02574	3.9542	0.000129
ARL4D	0.4928	5.26E-05	0.5606	0.000166
KCNIP2	0.7331	6.5E-06	0.7615	0.000196
GHR	0.539	0.01034	0.7602	0.000199
SLC6A2	0.922	0.01856	1.3564	0.000354
ENHO	1.828	0.02787	2.636	0.000424
PYROXD2	0.5938	0.01438	0.8131	0.000456
SMIM5	0.5092	0.0304	0.8763	0.00047
SPSB2	0.6099	0.000928	0.6387	0.000485
KATNIP	0.3079	0.01604	0.45	0.000528
AHSG	0.2763	0.03488	0.4162	0.000642
MOB3B	0.4241	7.6E-05	0.309	0.000917
CCDC88C	0.3632	0.03338	0.5784	0.000967
LRRC24	0.4209	0.00215	0.4385	0.001086
MMP3	0.7653	0.03257	0.8914	0.001123
PPM1J	0.8738	0.007501	1.0356	0.001302
RAB17	2.416	0.003329	2.4199	0.00169
GALNT16	0.6136	0.009128	0.7364	0.001694
COMMD4	0.3781	0.000258	0.3222	0.001993
ADAMTS7	0.3756	0.02824	0.4551	0.002209
COQ4	0.2853	0.01798	0.3856	0.002346
SKIDA1	0.3787	0.04926	0.5874	0.002386
PADI6	2.026	0.04025	2.8021	0.002484
PADI1	1.579	0.03082	2.0931	0.002705
PAOX	0.5574	0.03243	0.6643	0.002918
ATP1A2	0.4252	0.01092	0.4816	0.003002
SYNPO2	0.2673	0.02937	0.3492	0.003202
IGFBP5	0.4214	0.0187	0.6159	0.003361
KIF5A	0.3012	0.007579	0.3722	0.003483
LTK	0.3741	0.0239	0.3886	0.004203
SELENBP1	0.3021	0.007937	0.3028	0.00438
ST8SIA4	0.3976	0.007733	0.3402	0.004629
PLCL1	0.7396	0.03067	0.8333	0.004678
TTYH1	1.489	0.00281	1.4361	0.004736
SUPT3H	0.3618	0.006472	0.4405	0.004913
PRRT2	0.3366	0.005384	0.3997	0.004923
SNTG1	0.3655	0.04424	0.5506	0.005016
TMEM121	1.42	0.009969	1.4673	0.005234
ASMTL	0.4194	0.009333	0.4142	0.007209
NAV3	0.6728	0.01112	0.7192	0.00785
ELL3	0.4011	0.000384	0.2994	0.007905
TNXB	0.3299	0.01272	0.4135	0.00797
SOCS1	1.06	0.01314	1.0848	0.009183
CHAC1	0.3005	0.04232	0.377	0.009252
TMEM177	0.41	0.02981	0.4394	0.01044
CDK20	0.2641	0.01811	0.314	0.01066
TTLL8	2.991	0.008676	2.8431	0.01292
DUSP26	1.328	0.006736	0.9878	0.01376
ARFGEF3	0.3382	0.02328	0.4053	0.0141
CPZ	2.3963	0.0215	2.538	0.01441
CFAP299	1.789	0.01372	1.68	0.01811
RBM20	0.4028	0.04249	0.4435	0.0229

MFSD13A	0.3889	0.002096	0.2841	0.02491
BRD3OS	0.3615	0.01961	0.3784	0.02597
KLF11	0.3688	0.008912	0.3285	0.02853
TELO2	0.3529	0.004926	0.2852	0.02854
IL18R1	2.89	0.01266	1.2627	0.03528
DOCK3	0.4411	0.0169	0.4615	0.04097
GPR21	1.2508	3E-08	0.3455	0.0411
PDE8B	0.3995	0.04466	0.4866	0.04292
ZNF599	0.6075	0.02021	0.6338	0.04539
PCDH18	0.6346	0.000515	0.4703	0.04918

Table S3. Metabolomics DAMs (n = 6 independent IEC isolations). Selection criteria: FDR < 0.01, fold change (FC) > 1, and VIP > 1.

Name	pvalue	FDR	vip
5'-S-Methyl-5'-thioadenosine	9.85E-20	2.04E-15	2.306353
Palmitoyl ethanolamide	3.21E-07	9.65E-05	2.164377
5,5-dimethyl-2-{[(2-phenylacetyl)amino]methyl}-1,3-thiazolane-4-carboxylic acid	2.62E-05	0.002815	2.019753
N-Benzoyl-2-methoxy-N-methylphenylalanine	4.39E-05	0.004223	2.045795
Rhodamine 110	5.05E-06	0.000779	2.083022
N-((2-Hydroxy-3-oxo-2-(pent-2-en-1-yl)cyclopentyl)acetyl)iso-leucine	2.66E-08	1.12E-05	2.211014
19(R)-hydroxy Prostaglandin E2	6.23E-07	0.000156	2.135458
(4E)-2-(acetyloxy)-7-hydroxy-6-methoxy-7-(6-oxo-3,6-dihydro-2H-pyran-2-yl)hept-4-en-3-yl acetate	2.90E-08	1.20E-05	2.196974
2-[4-(2-Phenyl-2-propanyl)phenoxy]acetohydrazide	6.39E-05	0.005507	2.054026
Ouabain	6.43E-05	0.005507	2.048045
(2'R,3R,4a'S,5R,8a'S)-5-(3-Furyl)-2',5'-dimethyl-4',4a',8',8a'-tetra-hydro-2'H-spiro[furan-3,1'-naphthalene]-4,7'(3'H,5H)-dione	1.89E-05	0.002177	2.08985
Cortisone	1.51E-05	0.001781	2.064902
4-Hydroxybenzaldehyde	1.16E-05	0.001491	2.265296
Nicotinic acid	1.25E-08	5.75E-06	2.285071
Adenine	3.47E-12	5.13E-09	2.279215
Hypoxanthin	1.49E-05	0.001781	2.150762
Hypoxanthine	2.66E-05	0.00284	2.101756
acetylindole	3.70E-07	0.000108	2.291171
5-Methoxy-2-nitroaniline	0.000141475	0.009399	1.924898
(2,5-Dimethyl-1,3-thiazol-4-yl)acetic acid	2.74E-11	2.99E-08	2.285376
(5-Oxo-1-propyl-2-pyrrolidinyl)acetic acid	4.91E-06	0.000779	2.014057
N-Methyl-L-asparagine	1.30E-06	0.000268	2.123934
5-Methoxy-3-indoleaceate	6.25E-12	8.64E-09	2.271796
Alanyl-Glutamine	5.92E-19	6.14E-15	2.320295
6-Benzylaminopurine	1.06E-13	2.75E-10	2.293026
1-(2-Amino-3-hydroxyphenyl)-ethanone sulfate	5.37E-09	2.93E-06	2.222175
N-Benzoylisoleucine	3.64E-05	0.003661	2.030947
4-[(4-Nitrophenyl)amino]-4-oxobutanoic acid	5.84E-09	3.10E-06	2.217857
RZ2670000	9.95E-05	0.007653	2.100484
butralin	1.15E-18	7.93E-15	2.320721
Hyrtioerectine B	8.03E-12	9.80E-09	2.37575
8-hydroxy-11-(hydroxymethyl)-1,5,11-trimethyltricy-clo[6.2.1.0,]undec-2-en-9-one	1.72E-07	5.64E-05	2.454295
Muramic acid	7.56E-05	0.006296	1.984953
(5alpha,8beta,10alpha)-9(12)-Capnellene-5,8,10-triol	2.50E-06	0.000455	2.208259
3C-P	1.54E-07	5.43E-05	2.130409
D-(+)-Pyroglutamic Acid	0.0001335	0.00909	1.941812
3-[1-(2-Carboxyethyl)-1H-benzo[d]imidazol-2-yl]propanoic acid	2.24E-05	0.002477	2.034595
4-Acetamido-N-(4-nitrophenyl)butanamide	4.72E-16	2.45E-12	2.30015
Adenosine	8.55E-13	1.61E-09	2.316582
PHA-543613	0.000133376	0.00909	2.010796
Naptalam	7.80E-07	0.00018	2.153257
D-(-)-Luciferin	7.02E-08	2.69E-05	2.191451

2-[(3S)-1-(1-Methyl-4-piperidinyl)-3-pyrrolidinyl]-1H-benzimid-azole	0.000100062	0.007653	1.870347
Hydroxyanigorufone	1.29E-05	0.001609	2.070059
inosine	4.06E-09	2.34E-06	2.320803
N(6),O(2)-Dimethyladenosine	8.96E-07	0.0002	2.171461
